# MicroRNAs and oncogenic transcriptional regulatory networks controlling metabolic reprogramming in cancers

**DOI:** 10.1016/j.csbj.2016.05.005

**Published:** 2016-06-04

**Authors:** Pannapa Pinweha, Khanti Rattanapornsompong, Varodom Charoensawan, Sarawut Jitrapakdee

**Affiliations:** a Department of Biochemistry, Faculty of Science, Mahidol University, Bangkok 10400, Thailand; b Integrative Computational BioScience (ICBS) Center, Mahidol University, Nakhon Pathom 73170, Thailand

**Keywords:** ACC, acetyl-CoA carboxylase, ACL, ATP-citrate lyase, BRCA1, breast cancer type 1 susceptibility protein, c-MYC, V-myc avian myelocytomatosis viral oncogene homolog, FAS, fatty acid synthase, FH, fumarate hydratase, G6PD, glucose-6-phosphate dehydrogenase, GDH, glutamate dehydrogenase, GLS, glutaminase, GLUT, glucose transporter, HK, hexokinase, 2-HG, 2-hydroxyglutarate, HIF1α, hypoxia inducible factor 1α, IDH, isocitrate dehydrogenase, miR/miRNA, LDH, lactate dehydrogenase micro RNA, p53, tumor protein p53, PEP, phosphoenolpyruvate, MCT, monocarboxylic acid transporter, ME, malic enzyme, PEPCK, phosphoenolpyruvate carboxykinase, PFK, phosphofructokinase, PHGDH, phosphoglycerate dehydrogenase, PGK, phosphoglycerate kinase (PGK), PSAT, phosphoserine aminotransferase, PSPH, phosphoserine phosphatase, PKM, muscle-pyruvate kinase, PDH, pyruvate dehydrogenase, PC, pyruvate carboxylase, PDK, pyruvate dehydrogenase kinase, PPP, pentose phosphate pathway, SDH, succinate dehydrogenase, SHMT, serine hydroxymethyl transferase, SREBP1, sterol regulatory element binding protein 1, TCA, tricarboxylic acid, TFs, transcription factors, Cancer, Metabolism, MicroRNA, Oncogene, Transcriptional regulation network

## Abstract

Altered cellular metabolism is a fundamental adaptation of cancer during rapid proliferation as a result of growth factor overstimulation. We review different pathways involving metabolic alterations in cancers including aerobic glycolysis, pentose phosphate pathway, *de novo* fatty acid synthesis, and serine and glycine metabolism. Although oncoproteins, c-MYC, HIF1α and p53 are the major drivers of this metabolic reprogramming, post-transcriptional regulation by microRNAs (miR) also plays an important role in finely adjusting the requirement of the key metabolic enzymes underlying this metabolic reprogramming. We also combine the literature data on the miRNAs that potentially regulate 40 metabolic enzymes responsible for metabolic reprogramming in cancers, with additional miRs from computational prediction. Our analyses show that: (1) a metabolic enzyme is frequently regulated by multiple miRs, (2) confidence scores from prediction algorithms might be useful to help narrow down functional miR-mRNA interaction, which might be worth further experimental validation. By combining known and predicted interactions of oncogenic transcription factors (TFs) (c-MYC, HIF1α and p53), sterol regulatory element binding protein 1 (SREBP1), 40 metabolic enzymes, and regulatory miRs we have established one of the first reference maps for miRs and oncogenic TFs that regulate metabolic reprogramming in cancers. The combined network shows that glycolytic enzymes are linked to miRs via p53, c-MYC, HIF1α, whereas the genes in serine, glycine and one carbon metabolism are regulated via the c-MYC, as well as other regulatory organization that cannot be observed by investigating individual miRs, TFs, and target genes.

## 1 Overall metabolic reprograming in cancers

**Fig. 1:**
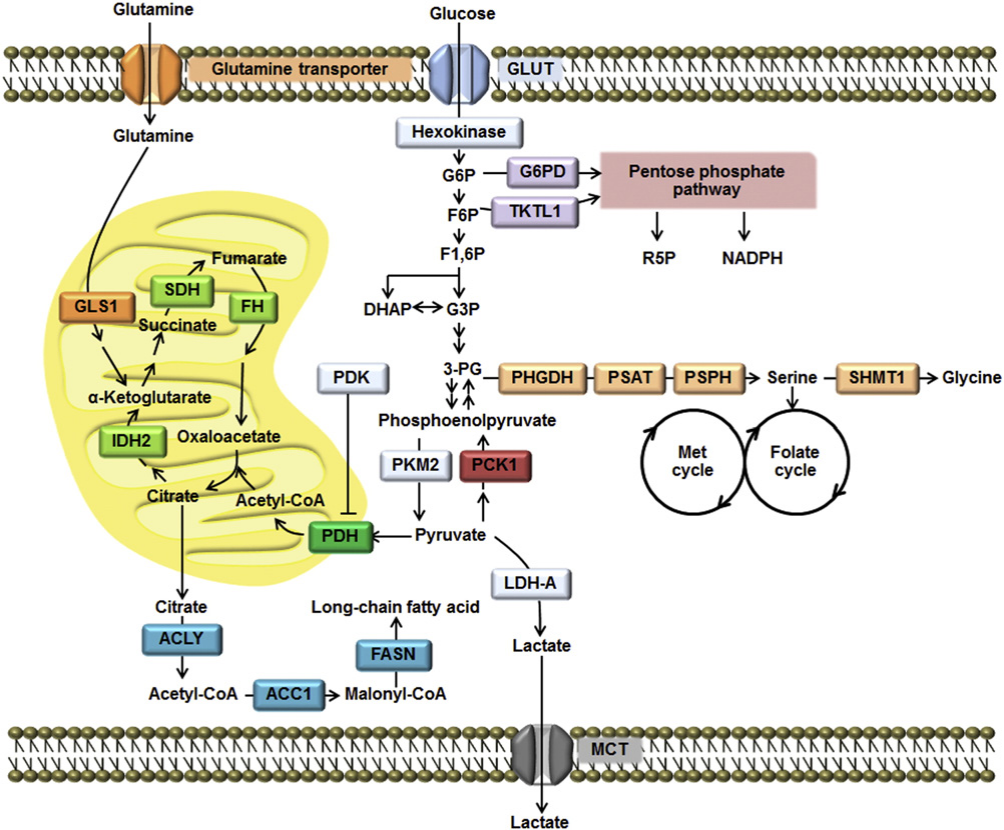
Metabolic pathways in cancers. Glucose and glutamine are two major carbon sources that are metabolized through these biochemical pathways.

In response to overstimulation of growth factor signaling, cancer cells reprogram their metabolism in order to accommodate a high demand for macromolecules during rapid proliferation [1], [2], [3], [4]. The hallmark of the above metabolic reprograming is the shift from oxidative phosphorylation to aerobic glycolysis, known as the “Warburg effect” [5]. This phenomenon provides some advantages to the tumors because aerobic glycolysis allows them to survive under hypoxic conditions, while an acidic environment selects a highly aggressive population of cancers to survive and metastasize to distal tissues or organs [3], [6]. Cancers are also highly anabolic because they require lipids, protein and nucleic acids as constituents of the structural components of the newly divided cells [2]. This highly anabolic phenotype is partly attributed to the Warburg effect because inhibition of pyruvate entering into the mitochondria results in the redirection of glycolytic intermediates to the pentose phosphate pathway (PPP), which provides biosynthetic precursors for nucleotides and lipids [4]. Furthermore, mitochondrial metabolism of cancers is also reprogrammed toward cataplerosis where substantial amounts of tricarboxylic acid (TCA) cycle intermediates are used as the biosynthetic precursors of lipids and amino acids [2]. Therefore, it is not surprising to see up-regulate expression of key enzymes that catalyze the above biosynthetic pathways in several types of cancers. Fig. 1 shows the overall metabolic reprogramming pathways in cancers together with the key regulatory enzymes.

Here we review the altered metabolic pathways and the relevant enzymes in cancers inferred from experimental and computational based data [7], [8], [9]. We also review the oncogenic transcription factors (TFs) and miRNAs that regulate those metabolic pathways. In addition, using known and predicted miRNA-target gene interaction, we establish and analyze the network of oncogenic miRNA-metabolic target gene networks that interplay and regulate metabolic reprograming in cancers.

### 1.1 miRNAs regulate metabolic pathways

Post-transcriptional regulation by microRNAs (miRNAs) has long been known as a mechanism to silence gene expression. miRNAs are short double stranded RNAs, comprising 15–25 nucleotides. They are first transcribed in the nucleus as the primary miRNAs, consisting of multiple stem loop structures, which are then subsequently digested to precursor miRNAs (pre-miRNAs) by Drosha, an RNase III family enzyme [10]. Pre-miRNAs are then transported to the cytoplasm where the hairpin structure is further removed by a dicer enzyme, yielding approximately 21 base pairs miRNA duplex. The miRNA duplex is subsequently incorporated in the Argonaute protein which digests one strand of the duplex miRNA, generating a single stranded miRNA. This single stranded miRNA is further brought to their target mRNAs by an RNA-induced silencing (RISC) complex. Binding of single stranded miRNAs to their targets is mediated by hybridization of 7–8 nucleotides of the miRNAs (known as seed match) to their complementary nucleotides in the 3′-untranslated regions of their targets. Such hybridization results in translational inhibition or degradation of target mRNAs, thus providing a means to inhibit gene expression. Furthermore, one miRNA can bind to more than one species of mRNA targets due to a non-stringent hybridization of the seed match region, allowing simultaneous down-regulation of multiple target mRNAs. In the same way, multiple species of miRNAs can bind to the same mRNA targets and enhance translational inhibition [11]. It is estimated that 45,000 miRNA target sites are found in the human genome, and these miRNAs control expression of up to 60% of human genes [12].

miRNAs are implicated in the regulation of various biological processes. Biochemically, miRNAs also regulate cellular metabolism either directly by targeting key enzymes of metabolic pathways or indirectly by modulating the expression of important transcription factors. Multiple studies have revealed that the altered metabolic pathways in cancers are tightly regulated by miRNAs [13]. In the first half of the review, we describe the metabolic pathways and key enzymes that are altered in various cancers and regulated by miRNAs. This will be followed by the second half on the regulatory networks between metabolic enzymes, regulatory miRNAs and oncogenic transcription factors.

### 1.2 Glycolytic and pentose phosphate pathways

The Warburg effect is a primary event of metabolic reprogramming during tumorigenesis. This effect includes induced expression of enzymes such as GLUT1, hexokinase 2 (HK2), phosphofructokinase 2 (PFK2) and pyruvate dehydrogenase kinase 1 (PDK1) [3]. Up-regulation of the expression of the first three targets results in a rapid uptake of glucose and increased glycolytic rate, while increased expression of PDK1 inactivates pyruvate dehydrogenase, restricting the conversion of pyruvate to acetyl-CoA in the mitochondria and thus uncoupling glycolysis from subsequent mitochondrial oxidation. Increased expression of lactate dehydrogenase and monocarboxylic acid transporter 4 (MCT4) further sequesters pyruvate toward lactate production, lowering the pH of the extracellular environment [14]. The muscle-specific pyruvate kinase M (PKM) isoform has also been implicated in metabolic reprogramming in certain cancers [15]. PKM exists in two isoforms, PKM1 and PKM2 that have arisen from alternative splicing of exons 9 and 10 [16]. The activities of these two enzymes are determined by their conformers. PKM1 has a tendency to form tetramers that possess high enzymatic activity while PKM2 shows relatively low activity due to its main conformer being dimers. PKM1 is the most abundant isoform in skeletal muscle while PKM2 is highly expressed during embryonic development. In many cancers, PKM2 is selectively expressed, resulting in the accumulation of phosphoenolpyruvate, and thus redirecting the flow of glycolytic intermediates toward the pentose phosphate pathway (PPP) [15]. This mechanism provides a great benefit for cancers because PPP provides the ribose-5-phosphate and NADPH required for the synthesis of nucleotides and fatty acids. PKM2 also plays a non-metabolic role in which it can act as a co-activator of TFs including HIF1α, STAT3, Oct4 and β-catinin which regulate expression of certain oncogenes [16], [17]. Therefore PKM2 switching can reprogram metabolic pathways and alter the program of gene expression in cancers.

In response to PKM2 activation or by other mechanisms, PPP activity has been reported to be elevated in many cancers [18]. Therefore it is not surprising to see up-regulation of key enzymes in this pathway including glucose-6-phosphate dehydrogenase (G6PD), 6-phosphogluconate dehydrogenase (6-PGD) and transketolase-like enzyme [19], [20], [21]. NADPH produced by PPP is also crucial for maintaining the proper glutathione-redox loop that cancers use to counter the reactive oxygen species formed especially during epithelial–mesenchymal-transition (EMT) or anoikis resistance [22], [23]. Inhibition of PPP via the use of specific enzyme inhibitors or siRNAs targeted to their corresponding enzymes retards growth and biosynthesis of lipid and nucleotides in many types of cancers [21], [24], [25].

### 1.3 Mitochondrial metabolism

The tricarboxylic acid cycle (TCA cycle) provides both catabolic and anabolic functions for living cells. In normal cells, the TCA cycle functions as a central oxidation hub where acetyl-CoA derived from oxidations of glucose, amino acids and fatty acids enters for complete oxidation. However in dividing cells or cancers, the TCA cycle is used as an anabolic hub because its intermediates are used as biosynthetic precursors of amino acids, nucleotides and lipids, in a process known as “cataplerosis” [26]. Mutations of certain TCA cycle enzymes such as isocitrate dehydrogenase (IDH), succinate dehydrogenase (SDH) and fumarate hydratase (FH) can contribute to tumorigenesis [27], [28]. In certain cancers especially glioma, mutations of the cytosolic (IDH1) or mitochondrial (IDH2) enzymes create a novel function in which they can further convert α-ketoglutarate to 2-hydroxyglutarate (2-HG) [29]. 2-HG is an oncometabolite because it acts as an inhibitor of α-ketoglutarate-dependent dioxygenase involved in DNA and histone demethylation. Inhibition of such a process can lead to tumorigenesis [2], [29]. Similarly, mutations of the genes encoding succinate dehydrogenase (SDH) and fumarate hydratase (FH) result in the accumulation of succinate or fumarate, respectively. These two metabolites are inhibitors of prolyl hydroxylase (PHD), which hydroxylates hypoxia-inducible factor 1α (HIF1α), resulting in its degradation by proteolysis. Therefore elevated levels of both metabolites stabilize HIF1α, activating glycolysis in cancers [27].

Cancers also require the replenishment of TCA cycle intermediates after their removal for biosynthetic purposes. In order to prevent a discontinuity in the supply of biosynthetic precursors, there is a biochemical pathway known as “anaplerosis” which is composed of two main reactions, glutaminolysis [30] and pyruvate carboxylation [31]. Glutaminolysis is the conversion of glutamine to glutamate by glutaminase (GLS) before glutamate is further converted to α-ketoglutarate in the TCA cycle by glutamate dehydrogenase. The second anaplerotic reaction is the carboxylation of pyruvate to oxaloacetate by pyruvate carboxylase (PC). Different cancers use these two different anaplerotic reactions to certain extents, to support biosynthesis by up-regulation of either or both enzymes during tumorigenesis [32], [33], [34], [35]. Inhibition of these two enzymes results in impaired growth of cancers accompanied with marked reduction in biosynthesis of lipids, nucleotides and amino acids [33], [34], [35], [36]. Recent studies show that a gluconeogenic enzyme, phosphoenolpyruvate carboxykinase (PEPCK) also plays an important role in supporting biosynthesis of tumors [37], [38], [39]. PEPCK catalyzes a further conversion of oxaloacetate to phosphoenolpyruvate (PEP). This enzyme occurs in two isoforms: the cytosolic (PEPCK1 or PEPCK-C) and the mitochondrial (PEPCK2 or PEPCK-M) isoforms. Colon cancer, for instance, uses PEPCK1 [39] while non-small cell lung cancer uses PEPCK2 [37], [38] to supply PEP to support their growth, respectively. However, PEP formed by both enzymes is not only converted to glucose but also used locally as a biosynthetic precursor of serine and glycine. Furthermore, elevated levels of PEP also drive the flow of the upstream glycolytic intermediate glucose-6-phosphate to enter the PPP for the synthesis of ribose sugar required for nucleotide synthesis [37], [39]. Interestingly, this function becomes more obvious when the nutrient that supports the growth of a tumor is shifted from glucose to glutamine [37], [39]. This adaptive mechanism enables cancers to grow and survive under glucose-limited conditions.

### 1.4 Amino acid synthesis

Amino acids serve as not only the building blocks of polypeptides, but also the precursors of nucleotides. As cancers require large amounts of proteins and nucleic acids, it is not surprising that up-regulation of key enzymes involved in biosynthesis of certain amino acids were observed in cancer cells. Serine and glycine are essential for synthesis of nucleotides as deprivations of these two amino acids endogenously or exogenously, retard growth of many cancers [40]. *De novo* synthesis of these two amino acids is started from 3-phosphoglycerate (3-PG), an intermediate in the glycolytic pathway. 3-PG is then converted to serine via a three-step reaction, in which 3-PG is first converted to 3-phosphohydroxypyruvate by phosphoglycerate dehydrogenase (PHGDH). 3-phosphohydroxypyruvate is further converted to serine by another two reactions catalyzed by phosphoserine aminotransferase (PSAT) and phosphoserine phosphatase (PSPH) [40]. As only 10% of 3-PG in the glycolytic pool enters serine and glycine biosynthesis, this seems paradoxical with such a high demand for both amino acids during the rapid proliferation of cancers. However, many cancers cope with this limitation via an aberrant activation of the serine biosynthetic pathway by increasing the copy number of the *PHGDH* gene or up-regulating its mRNA expression, resulting in much a higher rate of serine synthesis [41], [42]. Serine is further converted to glycine by the serine hydroxymethyl transferase (SHMT), a folate-dependent pathway [40]. SHMT is comprised of two isoforms, SHMT1 which is expressed in the cytoplasm whereas SHMT2 is expressed in mitochondria. It remains unclear about the functional redundancy of these two isoforms as inhibiting activity of either isoform or suppressing their expression retards growth in different cancer models [43], [44], [45]. Nevertheless, both SHMT1 and SHMT2 are associated with the folate cycle, which is involved in one-carbon metabolism including synthesis of methionine and nucleotides, and in histone methylation. Thus, disruption of both SHMT isoforms can potentially perturb these metabolic processes [40].

### 1.5 Lipid biosynthesis

Fatty acids especially in phospholipids are important components of the plasma membrane. In cancers, fatty acids are mainly synthesized through the *de novo* pathway either from glucose or glutamine via glycolysis or glutaminolysis, respectively. However, the latter pathway plays a more significant role in this process [46]. As mentioned earlier, glutamine enters the TCA cycle via glutamate before being converted to α-ketoglutarate by glutamate dehydrogenase. This glutaminolytic flux increases TCA cycle intermediate pools, enabling citrate to leave the mitochondria to enter the cytosol where it is decarboxylated to oxaloacetate and acetyl-CoA by the ATP-citrate lyase (ACL). It has been reported that ACL expression and activity are elevated in many cancers. Thus, inhibition of its activity impairs lipid synthesis and is accompanied by reduced cell growth and survival [47], [48]. The cytosolic acetyl-CoA then serves as a precursor for long chain acyl-CoA synthesis, which is highly regulated by two enzymes, acetyl-CoA carboxylase 1 (ACC1) and fatty acid synthase (FAS). ACC1 catalyzes the carboxylation of acetyl-CoA to form malonyl-CoA, a building block that donates two carbon units for fatty acid synthesis. ACC1 activity can be modulated by a reversible phosphorylation. Among other kinases, the AMP-activated protein kinase (AMP) can phosphorylate ACC1, transforming it into an inactive form while protein phosphatase 1 dephosphorylates ACC1 back to an active form [49]. The phosphorylated ACC1 is subjected to a second mode of regulation through interaction with a DNA repair protein, BRCA1 which is highly expressed in breast tissue [49]. This interaction sequesters phosphorylated ACC1 from being dephosphorylated thereby blocking fatty acid synthesis [50], [51]. A high incidence of the oncogene BRCA1 mutations is associated with breast cancer because these mutations not only result in the loss of BRCA1 function as a DNA repair protein but also perturbs its interaction with phosphorylated ACC1, freeing it to be dephosphorylated and subsequently stimulate lipogenesis in breast tissue [51], [52]. ACC1 is one of the anti-cancer drug targets because inhibiting its expression or activity induces apoptosis in many cancers [53], [54], [55]. FAS has also been reported to be aberrantly activated in many cancers [56], [57], [58]. Like ACC1, inhibition of FAS expression or activity markedly reduces cancer growth [52], [59], [60].

### 1.6 Metabolic pathway crosstalk contributing to tumorigenesis

Although the crosstalk of signaling pathway is well implicated in tumorigenesis [61], only a few examples of metabolic pathway crosstalk are reported in certain cancers. As mentioned earlier, accumulation of succinate in cancers bearing mutations of succinate dehydrogenase gene not only results in the inactivation of HIF1α, contributing to Warburg effect but this also promotes tumorigenesis by attenuating the production of glutathione, an important redox protein which functions in detoxifying reactive oxygen species (ROS). Several cancers overproduce ROS in order to enhance PI3K, MAPK and NF-κB signaling pathways that support cellular proliferation [1]. Elevated levels of fumarate are found to react with glutathione to form succinated glutathione thereby reducing the NADP/NADPH-couple regeneration system required to eliminate ROS [62]. Similar reduction of glutathione levels was also observed in glioma bearing IDH1 or IDH2 mutation which accumulates 2-HG, suggesting that this oncometabolite may support ROS formation through attenuating the anti-oxidant system [63]. Warburg effect may also enhance tumorigenesis via conversion of fructose-6-phosphate into hexosamine biosynthetic pathway, yielding O-linked N-acetylglucosamine that can enhance mitogenic signaling pathway [64].

### 1.7 Coordinate regulation of metabolic reprogramming in cancers by oncogenic transcription factors

Having outlined different pathways and mechanisms of metabolic reprogramming in cancers, an important question remains: what controls this metabolic reprogramming in cancers? Three major TFs, namely c-MYC, hypoxia inducible factor 1α (HIF1α) and p53 are responsible for simultaneous up-regulation of the above key metabolic enzymes [65]. Aberrant expression of c-MYC is observed in more than 50% of cancers and it is one of the most amplified oncogenes. The c-MYC regulates various biological processes including proliferation, apoptosis and metabolic reprogramming [66]. Elevated c-MYC levels in turn bind to its target gene promoters, which contain a canonical E-box (CANNTG) element, resulting in increased mRNA transcripts. In normal situations, c-MYC expression is tightly regulated i.e., its expression is high during cell division but rapidly declines during cell cycle arrest [67]. In situations of metabolic alterations, c-MYC targets expression of genes encoding GLUT1, HK2, PDK1 and GLS1 [65], [66], [68].

The hypoxia-inducible factor (HIF1α), another key oncogenic TF, is functionally coordinated with c-MYC in controlling metabolic reprogramming in cancers [69]. HIF1α exists into two forms: the non-hydroxylated and the hydroxylated forms. In the presence of oxygen, HIF1α undergoes hydroxylation by prolyl hydroxylase, making it prone to proteolysis. However, when oxygen concentration is low, HIF1α escapes hydroxylation, allowing it to enter to the nucleus where it is hetero-dimerized with HIF1β and binds to the hypoxia-responsive element (HRE) in the promoters of genes whose products are involved in angiogenesis and metabolism [3]. HIF1α's metabolic targets appear to overlap with those of c-MYC, including GLUT1, GLUT3, HK1, HK2, aldolase A, phosphoglycerate kinase (PGK), lactate dehydrogenase (LDH), monocarboxylic acid transporter 4 (MCT4), PDK1 and PKM2 [65], [70].

Unlike c-MYC and HIF1α, p53 functions as a tumor suppressor protein. Expression of p53 is highly regulated as its expression is essentially low in unstressed cells whereas it becomes highly expressed under stress conditions such as oxidative damage, nutrient limitations and DNA damage [67]. De-regulation of p53 expression caused by mutations is associated with more than half of all cancers [71]. As a transcription factor, p53 binds to the promoter of other tumor suppressor genes such as those involved in cell cycle arrest, DNA repair, apoptosis and metabolism. In addition, p53 can regulate turnover of many proteins independently of transcription [67]. In regard to its regulatory roles on metabolism, p53 inhibits expression of GLUT1, GLUT3, GLUT4, phosphoglycerate mutase 1 (PGM 1), and thus blocking excessive entry of glucose through glycolytic flux [67], [72]. p53 inhibits expression of MCT1 and PDK2 while activates expression of PDH1α subunit of PDH complex thereby coupling glycolysis with oxidative phosphorylation [73]. The p53 also down-regulates biosynthesis by decreasing the activity and abundance of glucose-6-phosphate dehydrogenase (G6PD) [74] and decreasing expression of malic enzymes ME1 and ME2 [67], [73]. As these three enzymes provide NADPH for biosynthesis, reducing their expression or activities would favor oxidative rather than biosynthetic pathways. In addition to controlling pathways that provide NADPH, p53 can also regulate *de novo* fatty acid synthesis via down-regulating the expression of the sterol regulatory protein 1c (SREBP1c), which is a key transcriptional factor controlling expression of ACL and FAS genes [73]. Therefore, loss-of-function mutations of p53 in cancers literally shift their metabolic phenotype from an oxidative fate to aerobic glycolysis and anabolism. The p53 protein also targets degradation of PEPCK and G6Pase in non-small cell lung cancer [75], [76].

### 1.8 Expanding the repertoire of miRNA target of the alterative expressed metabolic genes in cancer using computational prediction

It has now become clear that many cellular genes including those encoding metabolic enzymes are regulated by miRNAs [13]. Several studies have identified regulatory miRNAs of the key enzymes responsible for metabolic reprogramming while some miRNAs regulate the expression of oncogenic TFs (e.g. c-MYC, HIF1α and p53), which in turn regulate expression of those metabolic enzymes. Despite an increasing number of studies on regulation of metabolic genes through miRNAs in cancers, it is clear that the list of studies on miRNA-regulated metabolic enzymes in cancers is nowhere close to the completion. Furthermore, it is still not known whether some key metabolic enzymes e.g. HK1, Aldolase, MCT4, SHMT2, ACC1, can be regulated by certain miRNAs. Thus, here we sought to explore the repertoire of miRNAs that target expression of key enzymes involved in metabolic reprogramming in cancers by combining known interactions from literature (Table 1) and computational prediction (Table S1, Table S2). One of the most important challenges of computational prediction of miRNA is the specificity of the prediction algorithms, which are known to give a large number of false positives. To this end, we examined whether the prediction miRNAs are consistent with the functional validation shown in Table 1, and the predicted miRNA-mRNA interactions that would potentially be worth following up experimentally.

The most frequently used algorithms and webtools currently available for miRNA prediction include miRanda–mirSVR [77], [78], DIANA-microT-CDS [79], TargetScan [80], [81], Pictar [82], miRDB [83], and RNA22 [84], which use common features such as seed match and sequence conservation across the species [85]. In brief, the seed match is a perfect pairing between miRNA and the 3′-UTR of mRNA targets, which usually starts at the 5′ end of miRNA at the positions 2 to 8. There are four main classes of canonical seed matches including (1) 6-mer (6 perfect nucleotide matches between miRNA at positions 2 to 7 and mRNA target), (2) 7mer-A1 (perfect match of miRNA at positions 2 to 7 with an A opposite position 1 of mRNA target), (3) 8-mer (perfect seed paring of miRNA at positions 2 to 8 with an A opposite position 1 of mRNA target) [86] and (4) 7mer-8mer (perfect match of miRNA at positions 2 to 8 and mRNA target) [87], [88]. However, these different seed matches do not reflect the degrees of gene expression suppression by miRNAs [89].

With an aim to explore other potential miRNAs that may regulate key metabolic enzymes listed in Table 1, we choose two widely-used miRNA prediction tools that utilize different features to predict miRNA of the target mRNAs of interest, TargetScan7.0 and miRanda–mirSVR. The former predicts the miRNAs targeting a given gene based on the seed match and sequence conservation across the species, whilst the latter uses free energy binding between miRNA and mRNA targets, and the site accessibility for miRNA target prophecy [85]. The context ++ scores and mirSVR scores were used as the parameters to indicate the confidence of predictions from the TargetScan7.0 and miRanda–mirSVR, respectively. The context ++ score is the sum of contribution from 14 features [81], such as site-type, 3′ pairing, the local AU content [89], target site abundance, seed-pairing stability [80]. The mirSVR scores, on the other hand, can also rank the empirical probability of down-regulation using supervised machine learning of mRNA expression changes as a result of specific microRNA transfection [78]. In short, the more negative context ++ scores and mirSVR scores from the predictions reflect the higher “likelihood” that the mRNA is targeted by miRNA, and thus down-regulated gene expression.

**Fig. 2:**
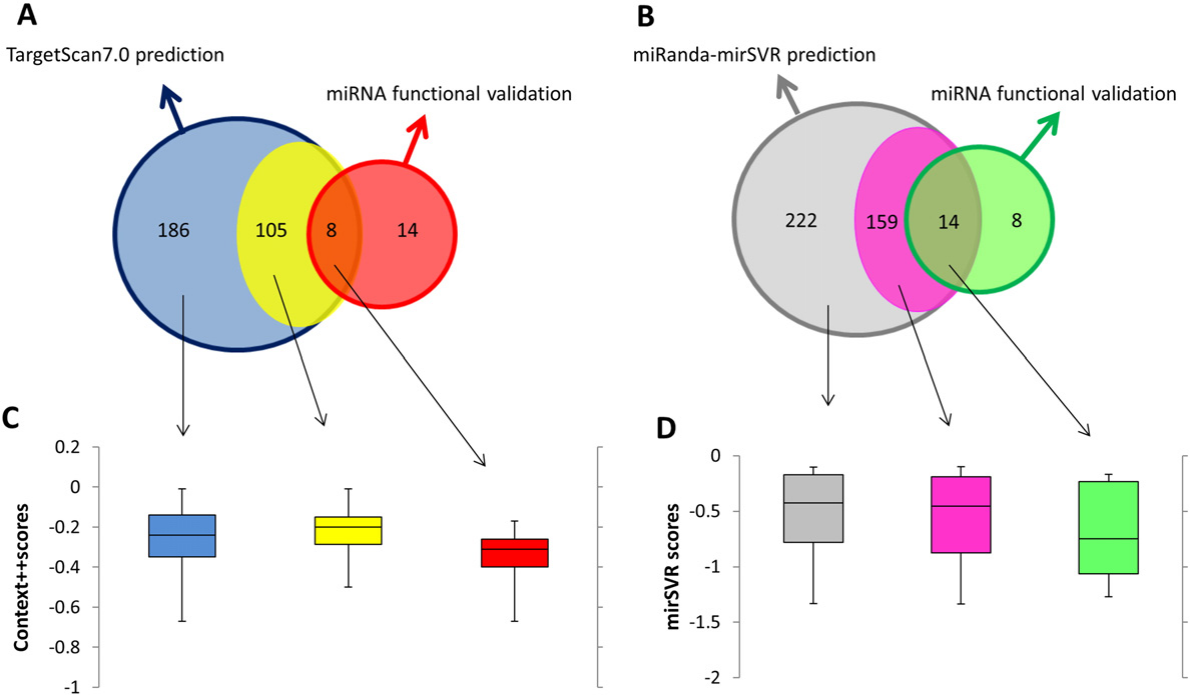
Venn diagrams and boxplots representing the association between miRNA prediction scores and their functional validation. The Venn diagrams of TargetScan7.0 (Fig. 2A) and miRanda–mirSVR (Fig. 2B) show the numbers of validated and predicted miRNAs that regulate metabolic enzymes in cancers. Boxplots illustrate the association of between context ++ scores (Fig. 2C) or miRanda–mirSVR scores (Fig 2D), and three miRNA groups: (1) experimentally validated miRNAs with prediction (2) miRNAs predicted to target metabolic enzymes with other verified miRNAs (3) the predicted miRNAs of altered metabolic enzymes whose functions have not been validated for any miRNA before.

As shown in Fig. 2A, TargetScan7.0 predicted that 40 metabolic enzymes shown in Table 1 are regulated by 299 miRNAs (blue circle). Sixteen out of 40 metabolic enzymes were predicted to be regulated by 113 miRNAs. However, only 8 out of these 113 miRNAs have been reported to functionally regulate expression of these enzymes, leaving the other 105 miRNAs (yellow) whose functional verification is yet to be elucidated. We also noted that there are 14 miRNAs (red) that have been experimentally verified to regulate this set of metabolic genes but elude prediction by TargetScan7.0, suggesting a considerable degree of false negatives. TargetScan7.0 also predicted 186 additional miRNAs that are likely to regulate another 24 metabolic enzymes, whose regulatory miRNAs have not been studied. The list of miRNAs that are predicted to regulate theses 40 metabolic enzymes can be found in Supplementary Table S1.

In a similar trend but not identical, miRanda–mirSVR predicted that there are 395 miRNAs that can potentially regulate these metabolic enzymes (Fig. 2B). One hundred and seventy three miRNAs were predicted to regulate 16 metabolic enzymes while the other 222 miRNAs (gray) were predicted to target another 24 metabolic enzymes which are currently unknown to be regulated by any miRNAs. Within those 16 metabolic enzymes regulated by 173 miRNAs, only 14 miRNAs were independently reported to regulate expression of these metabolic enzymes while the functional verifications of the other 159 miRNAs (pink) are yet to be elucidated. Similar to the TargetScan7.0 prediction but with fewer number of false negatives, eight additional miRNAs have been reported to functionally regulate expression of these 16 metabolic enzymes but were not detected by the miRanda–mirSVR prediction.

Due to the issues of sensitivity and specificity of miRNA prediction algorithms mentioned earlier, we generated boxplots of the context ++ scores (Fig. 2C) and mirSRV scores (Fig. 2D), in three miRNA groups: (1) experimentally verified miRNAs with prediction, (2) miRNAs predicted for target genes with other verified miRNAs, but their own functions are yet to be validated, and (3) the predicted miRNAs of metabolic enzymes whose functions have not be validated for any miRNA before (as outlined in the Venn diagrams). We did indeed observe a modest trend that the validated miRNAs have lower context ++ scores, than predicted miRNAs without validation; however, the number of miRNAs in each group is likely to be too small to give a statistical significant result. Similarly, the same can be said about the scores assigned to mirSVR prediction, indicating that confidence scores from the prediction might be useful as an extra indicator to extract the predicted miRNA that are likely to be “real” functional miRNAs, and would be worth further experimental validation.

### 1.9 MicroRNAs and oncogenic transcriptional regulatory networks

**Fig. 3:**
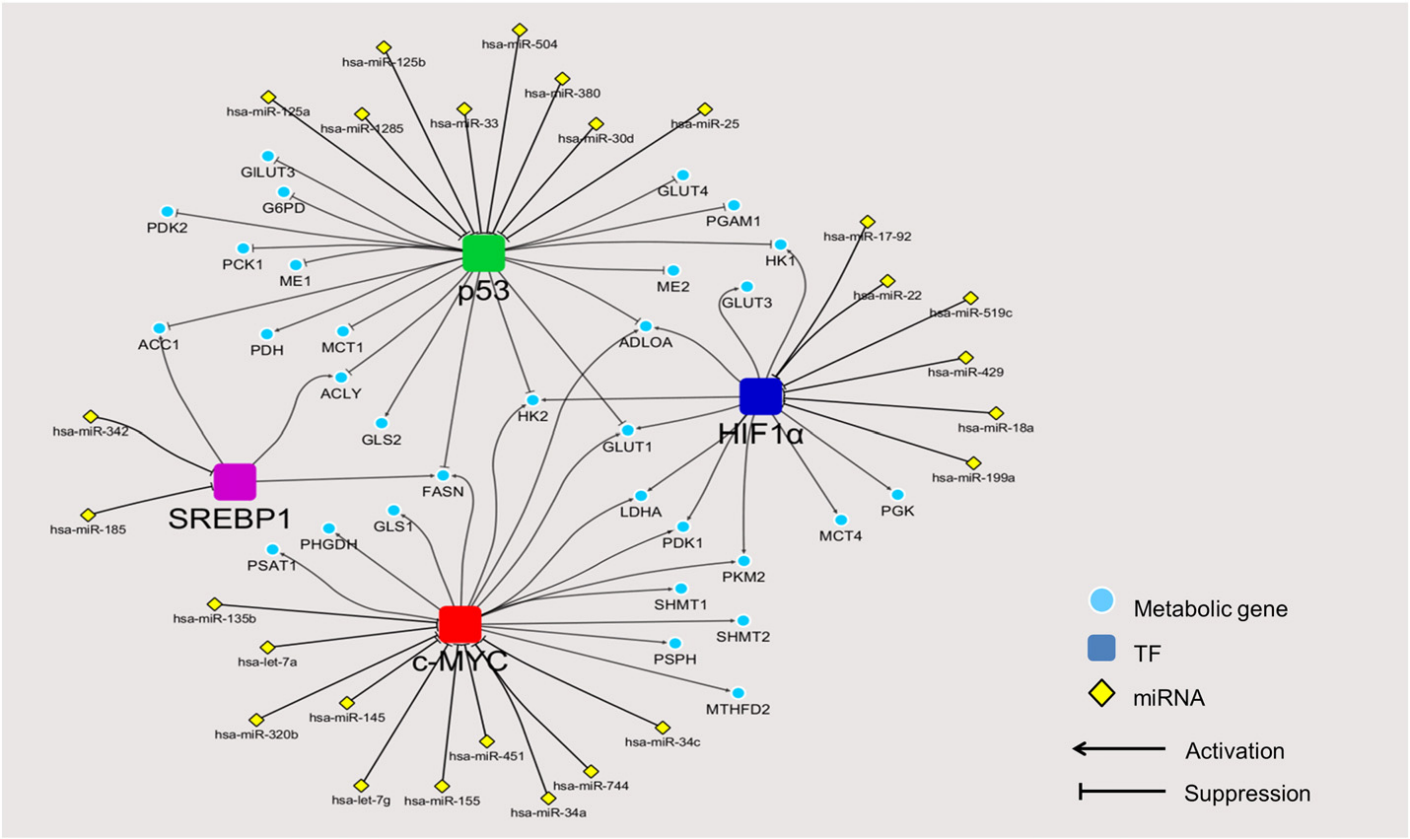
Regulatory network of experimentally verified miRNAs and oncogenic transcription factors controlling metabolic reprogramming in cancers. The figure shows the integration of experimentally validated regulatory network of TFs-cancer metabolic genes and miRNAs-TFs.

**Fig. 4:**
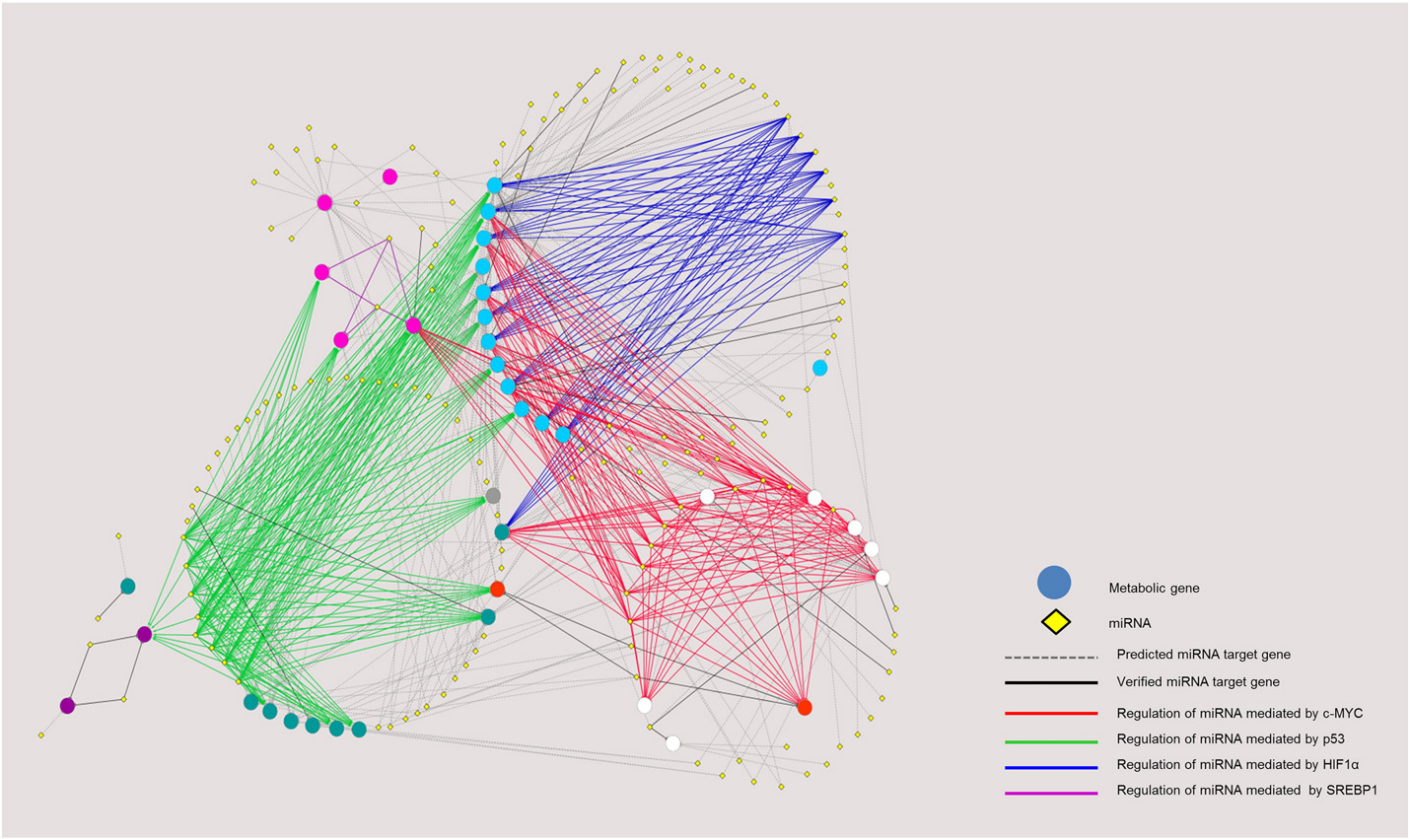
Regulatory network of miRNAs and oncogenic transcription factors controlling metabolic reprogramming in cancers. The figure shows direct and indirect miRNAs-metabolic genes interaction. The miRNAs that have already verified their regulatory function show in solid edges whereas the dash edges represent the overlap miRNAs from predictions only. In addition, direct interaction of experimentally verified miRNAs and gene targets are showed in black edges whilst the color edges (blue, green, red and purple) illustrate the interaction of miRNAs and cancer metabolic genes via oncogenic transcription factors. Blue edges represent the regulation of miRNA mediated HIF1α, green edges represent the regulation of miRNA mediated p53, red edges represent the regulation of miRNA mediated c-MYC and the purple edges represent the regulation of miRNA mediated SREBP1. The pale blue circle nodes show the anaerobic glycolytic genes, white circle nodes show genes in serine, glycine and one carbon metabolism, orange circle nodes show genes in glutaminolysis, pink circle nodes show genes in *de novo* fatty acid synthesis, purple circle nodes show genes in PPP pathways, gray circle node is PCK1 and the blue-green nodes show genes in TCA cycle. High resolution of the figure with complete labels can be found in Fig. S1.

To observe the overall interplay of oncogenic TFs, metabolic enzymes, and regulatory miRNAs, we combined the experimentally validated (Table 1), the experimentally validated miRNA-target data from miRTarBase [90] and predicted interactions (from the two algorithms as shown in Fig. 2) into a regulatory network of TFs-metabolic enzymes and miRNA-TFs using Cytoscape [91], as shown in Fig. 3, Fig. 4. Fig. 3 focuses on the known miRNAs that regulate expression of metabolic enzymes via controlling the expression of oncogenic TFs, whereas we expand the network to cover both validated and predicted miRNA-mRNA interactions in Fig. 4. The predicted interactions shown here are the overlaps of the two algorithms used: TargetScan7.0 and miRanda–mirSVR, shown as gray dashed edges, whereas the functional verified miRNA-gene targets from the Table 1 and miRTarBase database [90] are shown in black solid lines. The edges' colors (blue, red, green and purple) represent the miRNAs that regulate expression of metabolic enzymes through the expression of oncogenic TFs (HIF1α, c-MYC, p53, SREBP1, respectively), as in Fig. 3. The colors of node genes in Fig. 4 are classified by metabolic pathways: pale blue color for anaerobic glycolytic genes; white for enzymes involved in serine, glycine and one carbon metabolism; orange for GLS; blue-green nodes for enzymes in the TCA cycle; pink nodes for enzymes in the *de novo* fatty acid synthesis; gray nodes for gluconeogenic enzyme, and purple nodes for enzymes in the pentose phosphate pathway.

Overall, our miRNAs and oncogenic transcriptional regulatory network depicts individual “modules” of post-transcriptional regulation by miRNA via major drivers of metabolic reprogramming in cancers, acting as hubs that link multiple incoming miRNAs (yellow nodes, Fig. 3) that can bind and suppress transcription of these oncogenes, to their downstream metabolic gene targets (blue nodes). For instance, the expression of c-MYC (red node in Fig. 3, and interaction between miRNA and targeting metabolic genes via c-MYC are in red lines in Fig. 4) is regulated by let-7a in Burkitt Lymphoma [92], miR-145 in non-small cell lung cancer [93], let-7g and miR-744 in hepatocellular carcinoma cells [94], [95], miR-34 in prostate cancer cells [96], miR-135b in osteosarcoma cells [97], miR-155 in gastric carcinoma cells [98], miR-320b in colorectal cancer [99] and miR-451 in head and neck squamous cell carcinoma [100]. Suppression of these miRNAs contributes to overexpression of key metabolic enzymes in these tumors. Similarly, HIF1α ( dark blue node) expression is regulated by several miRNAs including miR-17-92 in lung cancer cells [101], miR-519c and miR-18a in breast and lung cancer cells [102], [103], miR-22 in colon cancer cells [104], miR-199a in non-small cell lung cancers [105] and miR-429 in human endothelial cells [106]. Ectopic expression of these miRNAs reduces the expression of vascular endothelial growth factor (VEGF), a crucial transcriptional target of HIF1α, thereby decreasing angiogenesis, a process of blood vessel formation required for tumor growth and metastasis [107]. Likewise, p53 (green node), a tumor suppressor is also post-transcriptionally regulated by several miRNAs such as miR-25 and miR-30d in myeloma cells [108], miR-125a in breast and hepatoblastoma cells [109], miR-125b in neuroblastoma and lung fibroblalst cells [110], miR-504 in breast and colon cancer cells [111], miR-1285 in neuroblastoma, hepatoblastoma and breast cancer cells [112], miR-33 in hematopoietic stem cells [113] and miR-380 in neuroblastoma cells [114]. Tight regulation of these miRNAs results in substantial expression of p53 which then leads to cell cycle arrest, thus maintaining cells in the non-proliferative state [115]. In contrast, an aberrant overexpression of these p53-target miRNAs results in the down-regulation of p53, causing malignancy. Because this group of miRNAs exerts its effect on the oncogenic transformation, they are generally now classified as the “oncomiR” miRNAs [116].

In addition to these three oncogenes, the sterol regulatory element binding protein (SREBP1, purple node) is also involved in metabolic reprogramming. SREBP1 is a TF that regulates expression of liver type-pyruvate kinase (PKL) and lipogenic enzymes, ACL, ACC and FAS, thus allowing *de novo* fatty acid synthesis from glucose in liver. Cancers also use SREBP1 to up-regulate expression of these lipogenic enzymes to support fatty acid synthesis. Similar to c-MYC, HIF1α and p53, expression of SREBP1 by itself is also regulated by miRNAs. miR-185 and miR-342 play important role in regulation of SREBP1 expression by direct binding to the 3′UTR of its mRNA [117]. Of particular interest, most lipogenic enzymes are co-regulated by more than one TF. For example ACL and ACC1 are regulated by both SREBP1 and p53, while FASN is regulated by SREBP1, p53 and c-MYC. Expression of HK1 is co-regulated by HIF1a and p53 while that of LDHA and PKM2 are co-regulated by HIF1α and c-MYC. GLU1, HK2 and ALDOA are the only three enzymes that are regulated by p53, HIF1α and c-MYC. Interestingly, the expression of certain miRNAs that regulate these metabolic enzymes can also be regulated by an oncogenic TFs. Gao et al. [118] showed that c-MYC indirectly regulates GLS expression in B lymphoma and prostate cancer by suppressing the expression of miR-23a/b that directly regulates the expression of GLS. Kim and coworkers also demonstrated that p53 blocks the expression of HK1, HK2, glucose-6-phosphate isomerase (GPI) and PDK1 by inducing miR-34a expression which in turn, down-regulates the expression of the above four enzymes [119].

**Fig. S1:**
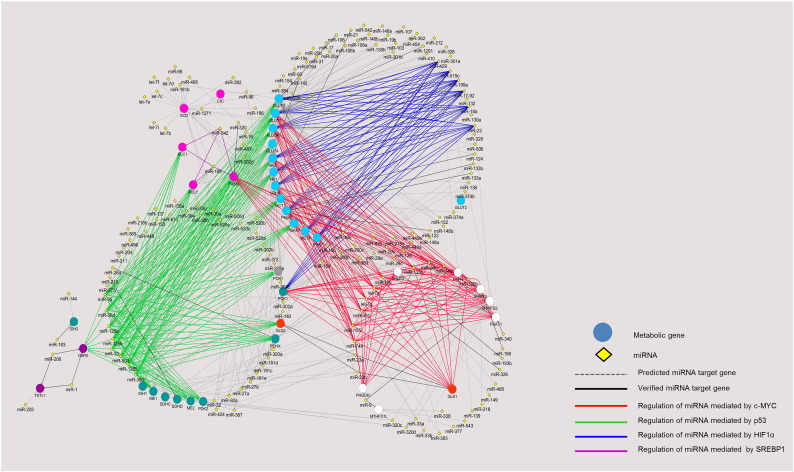
High-resolution map with complete labels of miRNA-TF-cancer metabolic gene regulatory network.

Looking at the expanded miRNA–mRNA interaction networks (Fig. 4), we observe a global overview of how metabolic genes involving cancer progression are regulated by miRNA through their direct interaction (black lines for validated interactions and gray lines for those predicted by TargetScan7.0 and miRanda–mirSVR), or through oncogenic TFs (colored edges). We have seen notable miRNAs such as miR-23a/b that directly control glutaminolysis, whereas the miR-1 and miR-206 are responsible for regulation of the PPP pathway genes, G6PD and TKTL1 [118], [120]. The overall network also highlights the “hub” miRNA. miR-429, a tumor suppressor that down-regulates almost all genes in anaerobic glycolytic pathway (e.g. GLUTs) via the oncogenic TF HIF1α. The anaerobic glycolytic genes themselves are also targeted by several other miRNAs such as miR-22, miR-199a, miR-17-92 via HIF1α (blue edges), miR-30d, miR-25, miR-125a/b, miR-1285 via p53 (green edges), and miR-451, miR-155, let-7a, let-7g via c-MYC (red edges). The network also demonstrates other relationships between metabolic pathways and miRNA regulation via TFs. For instance, three out of five genes in *de novo* fatty acid synthesis pathway (ACC1, ACLY, and FASN) share regulation by miRNAs via p53 and SREBP1. The genes in the serine, glycine and one carbon metabolism pathways (white nodes) heavily rely on the regulation of miRNAs via c-MYC. Post-transcriptional regulatory networks have demonstrated intricate regulation of metabolic genes by different miRNAs [13], [121], [122]. Here, we aim to provide a detailed regulatory network of metabolic genes under direct control of miRNAs, or oncogenic TFs regulated by miRNAs. The high resolution network with complete labels can be found in Supplementary material (Fig. S1 and Table S3). Such overall organization of metabolic gene expression regulation cannot be observed by studying miRNAs, TFs, and target genes individually. Saying that, we note that the current version of network relies on the accuracy of the two prediction algorithms used in this study. The known interactions taken from literature might also be biased toward well-characterized oncogenes such as p53 or c-MYC.

In conclusion, our review not only provides the current status of understanding metabolic reprogramming in cancers but also establishes the regulatory network of miRNA-oncogenic TF-cancer metabolic genes that would provide benefits for research guidance in this emerging field the future.

The following are the Supplementary data related to this article.

Table S1 Prediction of miRNAs that regulate metabolic enzymes by TargetScan7.0. Table S1

Table S2 Prediction of miRNAs that regulate metabolic enzymes by miRanda–mirSVR. Table S2

Table S3 miRNA-target gene interactions used to generate Fig. 4. Table S3

## Figures and Tables

**Table 1:** A list of 40 metabolic enzymes that are involved in metabolic reprogramming in cancers.

Enzyme	Full name	Gene	miRNA	References
*Aerobic glycolysis, Warburg effect*				
GLUT1	Glucose transporter 1	NM_006516	miR-1291 [123]	[124], [125], [126]
GLUT2	Glucose transporter 2	NM_000340	N/A	[124]
GLUT3	Glucose transporter 3	NM_006931	miR-195-5p [127], miR-106-5p [90], [128]	[124], [129], [125], [126]
GLUT4	Glucose transporter 4	NM_001042	N/A	[124], [130], [125]
HK1	Hexokinase1	NM_000188	N/A	[3]
HK2	Hexokinase2	NM_000189	miR-143 [131]^,^[132]	[133], [3]
Aldolase A	Aldolase A	NM_000034	N/A	[134]
PGAM1	Phosphoglycerate mutase 1	NM_002629	N/A	[135]
PKM2	Pyruvate kinase 2	NM_002654	miR-122, miR-133a, miR-133b,miR-326 [136], [137], [138]	[139], [140]
LDHA	Lactate dehydrogenase A	NM_005566	miR-21 [141]	[142], [143]
MCT1	Monocarboxylate transporter 1	NM_003051	miR-124 [144]	[145]
MCT4	Monocarboxylate transporter 4	NM_004696	N/A	[145], [146]

*Pentose phosphate pathway*				
G6PD	Glucose-6-phosphate dehydrogenase	NM_000402	miR-206, miR-1 [120]	[20]
TKTL1	Transketolase-like1	NM_012253	miR-206, miR-1 [120]	[19]

*Gluconeogenesis*				
PCK1	Phosphoenolpyruvate carboxykinase 1	NM_002591	N/A	[39]
PCK2	Phosphoenolpyruvate carboxykinase 2	NM_004563	N/A	[38], [37]

*Tricarboxylic acid (TCA) cycle*				
PDK1	Pyruvate dehydrogenase kinase 1	NM_002610	N/A	[147]
PDH	Pyruvate dehydrogenase	NM_003477	miR-26a [148]	[149]
IDH1	Isocitrate dehydrogenase 1	NM_005896	N/A	[28]
IDH2	Isocitrate dehydrogenase 2	NM_002168	miR-183 [150]	[28]
SDH-B	Succinate dehydrogenase complex iron sulfur subunit B	NM_003000	N/A	[27]
SDH-C	Succinate dehydrogenase complex subunit C	NM_003001	N/A	[27]
SDH-D	Succinate dehydrogenase complex subunit D	NM_003002	miR-210 [151]	[27]
FH	Fumarate hydratase	NM_000143	N/A	[27]
ME1	Malic enzyme 1	NM_002395	N/A	[152]

*Glutaminolysis*				
GLS1	Glutaminase 1	NM_014905	miR-23a, miR-23b [118]	[32]
GLS2	Glutaminase 2	NM_013267	miR-23a, miR-23b [118]	[153], [154]

*Serine, Glycine and one carbon metabolism*				
SHMT2	Serine hydroxymethyltransferase 2	NM_005412	miR-193b [90], [155]	[156]
SHMT1	Serine hydroxymethyltransferase 1	NM_004169	miR-198 [157]	[156]
MTHFD2	Methylenetetrahydrofolate dehydrogenase	NM_006636	miR-9 [158]	[156]
MTHFD1L	Methylenetetrahydrofolate dehydrogenase 1-like	NM_015440	miR-9 [158]	[156]
PHGDH	Phosphoglycerate dehydeogenase	NM_006623	N/A	[41]
PSAT1	Phosphoserine aminotransferase 1	NM_021154	miR-340 [159]	[160], [161]
PSPH	Phosphoserine phosphatase	NM_004577	N/A	[161]
GNMT	Glycine-N-methyltransferase	NM_018960	N/A	[162]

*de novo fatty acid synthesis*				
CIC	Citrate carrier	NM_005984	N/A	[163]
ACLY	ATP citrate lyase Y	NM_001096	N/A	[152], [164]
ACC1	Acetyl-CoA carboxylase 1	NM_198836	N/A	[152], [165]
FASN	Fatty acid synthase	NM_004104	miR-320 [166]	[58], [56], [57]
SCD	Stearoyl-CoA desaturase	NM_005063	N/A	[152]

Abbreviation: not available (N/A).
