# Supplementary material for: MicroRNAs and oncogenic transcriptional regulatory networks controlling metabolic reprogramming in cancers
Source: Comput Struct Biotechnol J. 2016 Jun 4;14:223–33. doi: 10.1016/j.csbj.2016.05.005 (PMC4915959; doi:10.1016/j.csbj.2016.05.005)
Supplement: Table S1 — Prediction of miRNAs that regulate metabolic enzymes by TargetScan7.0. [file mmc1.pdf]

Table S1.

Metabolic pathways	Target genes	miRNA family	context++ scores
Anearobic glycolysis	ALDOA	hsa-miR-122-5p	-0.53
Anearobic glycolysis	ALDOA	hsa-miR-1306-5p	-0.27
Anearobic glycolysis	ALDOA	hsa-miR-329-3p	-0.21
Anearobic glycolysis	ALDOA	hsa-miR-335-5p	-0.09
Anearobic glycolysis	ALDOA	hsa-miR-34a-5p	-0.66
Anearobic glycolysis	ALDOA	hsa-miR-34c-5p	-0.67
Anearobic glycolysis	ALDOA	hsa-miR-362-3p	-0.22
Anearobic glycolysis	ALDOA	hsa-miR-449a	-0.66
Anearobic glycolysis	ALDOA	hsa-miR-449b-5p	-0.67
Anearobic glycolysis	GLUT1	hsa-miR-130a-3p	-0.14
Anearobic glycolysis	GLUT1	hsa-miR-130b-3p	-0.16
Anearobic glycolysis	GLUT1	hsa-miR-132-3p	-0.22
Anearobic glycolysis	GLUT1	hsa-miR-140-5p	-0.23
Anearobic glycolysis	GLUT1	hsa-miR-142-3p.2	-0.16
Anearobic glycolysis	GLUT1	hsa-miR-148a-3p	-0.39
Anearobic glycolysis	GLUT1	hsa-miR-148b-3p	-0.39
Anearobic glycolysis	GLUT1	hsa-miR-152-3p	-0.39
Anearobic glycolysis	GLUT1	hsa-miR-19a-3p	-0.05
Anearobic glycolysis	GLUT1	hsa-miR-19b-3p	-0.05
Anearobic glycolysis	GLUT1	hsa-miR-212-3p	-0.2
Anearobic glycolysis	GLUT1	hsa-miR-22-3p	-0.29
Anearobic glycolysis	GLUT1	hsa-miR-301a-3p	-0.13
Anearobic glycolysis	GLUT1	hsa-miR-301b-3p	-0.13
Anearobic glycolysis	GLUT1	hsa-miR-3064-5p	-0.2
Anearobic glycolysis	GLUT1	hsa-miR-328-3p	-0.21
Anearobic glycolysis	GLUT1	hsa-miR-340-5p	-0.01
Anearobic glycolysis	GLUT1	hsa-miR-3666	-0.18
Anearobic glycolysis	GLUT1	hsa-miR-410-3p	-0.03
Anearobic glycolysis	GLUT1	hsa-miR-4295	-0.14

Anearobic glycolysis	GLUT1	hsa-miR-454-3p	-0.17
Anearobic glycolysis	GLUT1	hsa-miR-6504-5p	-0.2
Anearobic glycolysis	GLUT2	hsa-miR-299-5p	-0.32
Anearobic glycolysis	GLUT2	hsa-miR-374a-5p	-0.03
Anearobic glycolysis	GLUT2	hsa-miR-374b-5p	-0.03
Anearobic glycolysis	GLUT3	hsa-miR-103a-3p	-0.28
Anearobic glycolysis	GLUT3	hsa-miR-107	-0.28
Anearobic glycolysis	GLUT3	hsa-miR-1306-5p	-0.26
Anearobic glycolysis	GLUT3	hsa-miR-146a-5p	-0.19
Anearobic glycolysis	GLUT3	hsa-miR-146b-5p	-0.19
Anearobic glycolysis	GLUT3	hsa-miR-148a-3p	-0.19
Anearobic glycolysis	GLUT3	hsa-miR-148b-3p	-0.17
Anearobic glycolysis	GLUT3	hsa-miR-152-3p	-0.17
Anearobic glycolysis	GLUT3	hsa-miR-15a-5p	-0.38
Anearobic glycolysis	GLUT3	hsa-miR-15b-5p	-0.39
Anearobic glycolysis	GLUT3	hsa-miR-16-5p	-0.39
Anearobic glycolysis	GLUT3	hsa-miR-181a-5p	-0.22
Anearobic glycolysis	GLUT3	hsa-miR-181b-5p	-0.23
Anearobic glycolysis	GLUT3	hsa-miR-181c-5p	-0.22
Anearobic glycolysis	GLUT3	hsa-miR-181d-5p	-0.25
Anearobic glycolysis	GLUT3	hsa-miR-195-5p	-0.4
Anearobic glycolysis	GLUT3	hsa-miR-200b-3p	-0.08
Anearobic glycolysis	GLUT3	hsa-miR-200c-3p	-0.08
Anearobic glycolysis	GLUT3	hsa-miR-25-3p	-0.28
Anearobic glycolysis	GLUT3	hsa-miR-29a-3p	-0.3
Anearobic glycolysis	GLUT3	hsa-miR-29b-3p	-0.3
Anearobic glycolysis	GLUT3	hsa-miR-29c-3p	-0.3
Anearobic glycolysis	GLUT3	hsa-miR-32-5p	-0.25
Anearobic glycolysis	GLUT3	hsa-miR-363-3p	-0.27
Anearobic glycolysis	GLUT3	hsa-miR-367-3p	-0.25
Anearobic glycolysis	GLUT3	hsa-miR-378a-3p	-0.02
Anearobic glycolysis	GLUT3	hsa-miR-378b	-0.02

Anearobic glycolysis	GLUT3	hsa-miR-378c	-0.02
Anearobic glycolysis	GLUT3	hsa-miR-378d	-0.02
Anearobic glycolysis	GLUT3	hsa-miR-378e	-0.02
Anearobic glycolysis	GLUT3	hsa-miR-378f	-0.02
Anearobic glycolysis	GLUT3	hsa-miR-378h	-0.02
Anearobic glycolysis	GLUT3	hsa-miR-378i	-0.02
Anearobic glycolysis	GLUT3	hsa-miR-422a	-0.02
Anearobic glycolysis	GLUT3	hsa-miR-424-5p	-0.42
Anearobic glycolysis	GLUT3	hsa-miR-4262	-0.16
Anearobic glycolysis	GLUT3	hsa-miR-429	-0.08
Anearobic glycolysis	GLUT3	hsa-miR-497-5p	-0.4
Anearobic glycolysis	GLUT3	hsa-miR-503-5p	-0.35
Anearobic glycolysis	GLUT3	hsa-miR-542-3p	-0.14
Anearobic glycolysis	GLUT3	hsa-miR-6838-5p	-0.42
Anearobic glycolysis	GLUT3	hsa-miR-7153-5p	-0.18
Anearobic glycolysis	GLUT3	hsa-miR-92a-3p	-0.24
Anearobic glycolysis	GLUT3	hsa-miR-92b-3p	-0.24
Anearobic glycolysis	GLUT4	hsa-miR-106a-5p	-0.32
Anearobic glycolysis	GLUT4	hsa-miR-106b-5p	-0.3
Anearobic glycolysis	GLUT4	hsa-miR-1251-5p	-0.25
Anearobic glycolysis	GLUT4	hsa-miR-17-5p	-0.32
Anearobic glycolysis	GLUT4	hsa-miR-20a-5p	-0.29
Anearobic glycolysis	GLUT4	hsa-miR-20b-5p	-0.32
Anearobic glycolysis	GLUT4	hsa-miR-302a-3p	-0.21
Anearobic glycolysis	GLUT4	hsa-miR-302b-3p	-0.21
Anearobic glycolysis	GLUT4	hsa-miR-302c-3p.1	-0.21
Anearobic glycolysis	GLUT4	hsa-miR-302d-3p	-0.21
Anearobic glycolysis	GLUT4	hsa-miR-302e	-0.21
Anearobic glycolysis	GLUT4	hsa-miR-31-5p	-0.34
Anearobic glycolysis	GLUT4	hsa-miR-372-3p	-0.21
Anearobic glycolysis	GLUT4	hsa-miR-373-3p	-0.15
Anearobic glycolysis	GLUT4	hsa-miR-519d-3p	-0.3

Anearobic glycolysis	GLUT4	hsa-miR-520a-3p	-0.22
Anearobic glycolysis	GLUT4	hsa-miR-520b	-0.21
Anearobic glycolysis	GLUT4	hsa-miR-520c-3p	-0.21
Anearobic glycolysis	GLUT4	hsa-miR-520d-3p	-0.21
Anearobic glycolysis	GLUT4	hsa-miR-520e	-0.21
Anearobic glycolysis	GLUT4	hsa-miR-526b-3p	-0.23
Anearobic glycolysis	GLUT4	hsa-miR-93-5p	-0.31
Anearobic glycolysis	HK1	hsa-miR-138-5p	-0.41
Anearobic glycolysis	HK1	hsa-miR-302a-3p	-0.12
Anearobic glycolysis	HK1	hsa-miR-302b-3p	-0.16
Anearobic glycolysis	HK1	hsa-miR-302c-3p.1	-0.14
Anearobic glycolysis	HK1	hsa-miR-302d-3p	-0.13
Anearobic glycolysis	HK1	hsa-miR-302e	-0.14
Anearobic glycolysis	HK1	hsa-miR-346	-0.04
Anearobic glycolysis	HK1	hsa-miR-34a-5p	-0.19
Anearobic glycolysis	HK1	hsa-miR-34c-5p	-0.14
Anearobic glycolysis	HK1	hsa-miR-369-3p	-0.02
Anearobic glycolysis	HK1	hsa-miR-372-3p	-0.12
Anearobic glycolysis	HK1	hsa-miR-373-3p	-0.06
Anearobic glycolysis	HK1	hsa-miR-374c-5p	-0.02
Anearobic glycolysis	HK1	hsa-miR-449a	-0.13
Anearobic glycolysis	HK1	hsa-miR-449b-5p	-0.12
Anearobic glycolysis	HK1	hsa-miR-520a-3p	-0.12
Anearobic glycolysis	HK1	hsa-miR-520b	-0.12
Anearobic glycolysis	HK1	hsa-miR-520c-3p	-0.12
Anearobic glycolysis	HK1	hsa-miR-520d-3p	-0.12
Anearobic glycolysis	HK1	hsa-miR-520e	-0.12
Anearobic glycolysis	HK1	hsa-miR-655-3p	-0.03
Anearobic glycolysis	HK2	hsa-let-7a-5p	-0.18
Anearobic glycolysis	HK2	hsa-let-7b-5p	-0.18
Anearobic glycolysis	HK2	hsa-let-7c-5p	-0.18
Anearobic glycolysis	HK2	hsa-let-7d-5p	-0.2

Anearobic glycolysis	HK2	hsa-let-7e-5p	-0.18
Anearobic glycolysis	HK2	hsa-let-7f-5p	-0.18
Anearobic glycolysis	HK2	hsa-let-7g-5p	-0.18
Anearobic glycolysis	HK2	hsa-let-7i-5p	-0.18
Anearobic glycolysis	HK2	hsa-miR-125a-5p	-0.33
Anearobic glycolysis	HK2	hsa-miR-125b-5p	-0.29
Anearobic glycolysis	HK2	hsa-miR-143-3p	-0.17
Anearobic glycolysis	HK2	hsa-miR-151a-3p	-0.12
Anearobic glycolysis	HK2	hsa-miR-323a-3p	-0.09
Anearobic glycolysis	HK2	hsa-miR-330-3p	-0.02
Anearobic glycolysis	HK2	hsa-miR-4319	-0.26
Anearobic glycolysis	HK2	hsa-miR-4458	-0.2
Anearobic glycolysis	HK2	hsa-miR-4500	-0.22
Anearobic glycolysis	HK2	hsa-miR-4770	-0.17
Anearobic glycolysis	HK2	hsa-miR-493-5p	-0.16
Anearobic glycolysis	HK2	hsa-miR-6088	-0.17
Anearobic glycolysis	HK2	hsa-miR-9-5p	-0.15
Anearobic glycolysis	HK2	hsa-miR-98-5p	-0.18
Anearobic glycolysis	LDHA	hsa-miR-3167	-0.1
Anearobic glycolysis	LDHA	hsa-miR-323a-3p	-0.08
Anearobic glycolysis	LDHA	hsa-miR-33a-5p	-0.41
Anearobic glycolysis	LDHA	hsa-miR-33b-5p	-0.41
Anearobic glycolysis	LDHA	hsa-miR-34a-5p	-0.46
Anearobic glycolysis	LDHA	hsa-miR-34c-5p	-0.49
Anearobic glycolysis	LDHA	hsa-miR-383-5p.1	-0.29
Anearobic glycolysis	LDHA	hsa-miR-449a	-0.47
Anearobic glycolysis	LDHA	hsa-miR-449b-5p	-0.49
Anearobic glycolysis	LDHA	hsa-miR-501-3p	-0.19
Anearobic glycolysis	LDHA	hsa-miR-502-3p	-0.19
Anearobic glycolysis	LDHA	hsa-miR-670-3p	-0.06
Anearobic glycolysis	LDHA	hsa-miR-876-5p	-0.06
Anearobic glycolysis	MCT1	hsa-miR-124-3p.1	-0.58

Anearobic glycolysis	MCT1	hsa-miR-124-3p.2	-0.43
Anearobic glycolysis	MCT1	hsa-miR-128-3p	-0.17
Anearobic glycolysis	MCT1	hsa-miR-154-5p	-0.13
Anearobic glycolysis	MCT1	hsa-miR-216a-3p	-0.18
Anearobic glycolysis	MCT1	hsa-miR-29a-3p	-0.28
Anearobic glycolysis	MCT1	hsa-miR-29b-3p	-0.28
Anearobic glycolysis	MCT1	hsa-miR-29c-3p	-0.28
Anearobic glycolysis	MCT1	hsa-miR-342-3p	-0.28
Anearobic glycolysis	MCT1	hsa-miR-3681-3p	-0.16
Anearobic glycolysis	MCT1	hsa-miR-374a-5p	-0.06
Anearobic glycolysis	MCT1	hsa-miR-374b-5p	-0.06
Anearobic glycolysis	MCT1	hsa-miR-384	-0.12
Anearobic glycolysis	MCT1	hsa-miR-485-3p	-0.13
Anearobic glycolysis	MCT1	hsa-miR-506-3p	-0.36
Anearobic glycolysis	MCT1	hsa-miR-539-3p	-0.25
Anearobic glycolysis	MCT1	hsa-miR-582-5p	-0.3
Anearobic glycolysis	PGAM1	hsa-miR-135a-5p	-0.35
Anearobic glycolysis	PGAM1	hsa-miR-135b-5p	-0.35
Anearobic glycolysis	PGAM1	hsa-miR-1-3p	-0.14
Anearobic glycolysis	PGAM1	hsa-miR-206	-0.14
Anearobic glycolysis	PGAM1	hsa-miR-25-3p	-0.56
Anearobic glycolysis	PGAM1	hsa-miR-32-5p	-0.53
Anearobic glycolysis	PGAM1	hsa-miR-363-3p	-0.55
Anearobic glycolysis	PGAM1	hsa-miR-367-3p	-0.53
Anearobic glycolysis	PGAM1	hsa-miR-483-3p.2	-0.51
Anearobic glycolysis	PGAM1	hsa-miR-582-5p	-0.17
Anearobic glycolysis	PGAM1	hsa-miR-613	-0.14
Anearobic glycolysis	PGAM1	hsa-miR-92a-3p	-0.53
Anearobic glycolysis	PGAM1	hsa-miR-92b-3p	-0.53
Anearobic glycolysis	PKM2	hsa-miR-122-5p	-0.4
de novo fatty acid synthesis	ACLY	hsa-miR-22-3p	-0.22
de novo fatty acid synthesis	ACLY	hsa-miR-27a-3p	-0.14

de novo fatty acid synthesis	ACLY	hsa-miR-27b-3p	-0.13
de novo fatty acid synthesis	ACLY	hsa-miR-369-3p	-0.08
de novo fatty acid synthesis	ACLY	hsa-miR-374c-5p	-0.18
de novo fatty acid synthesis	ACLY	hsa-miR-655-3p	-0.18
de novo fatty acid synthesis	CIC	hsa-miR-10a-5p	-0.02
de novo fatty acid synthesis	CIC	hsa-miR-10b-5p	-0.02
de novo fatty acid synthesis	CIC	hsa-miR-124-3p.1	-0.16
de novo fatty acid synthesis	CIC	hsa-miR-1271-5p	-0.54
de novo fatty acid synthesis	CIC	hsa-miR-331-3p	-0.14
de novo fatty acid synthesis	CIC	hsa-miR-96-5p	-0.52
de novo fatty acid synthesis	FASN	hsa-miR-15a-5p	-0.29
de novo fatty acid synthesis	FASN	hsa-miR-15b-5p	-0.28
de novo fatty acid synthesis	FASN	hsa-miR-16-5p	-0.31
de novo fatty acid synthesis	FASN	hsa-miR-195-5p	-0.31
de novo fatty acid synthesis	FASN	hsa-miR-27a-3p	-0.04
de novo fatty acid synthesis	FASN	hsa-miR-27b-3p	-0.04
de novo fatty acid synthesis	FASN	hsa-miR-424-5p	-0.27
de novo fatty acid synthesis	FASN	hsa-miR-497-5p	-0.27
de novo fatty acid synthesis	FASN	hsa-miR-6838-5p	-0.27
de novo fatty acid synthesis	SCD	hsa-let-7a-5p	-0.36
de novo fatty acid synthesis	SCD	hsa-let-7b-5p	-0.35
de novo fatty acid synthesis	SCD	hsa-let-7c-5p	-0.36
de novo fatty acid synthesis	SCD	hsa-let-7d-5p	-0.36
de novo fatty acid synthesis	SCD	hsa-let-7e-5p	-0.36
de novo fatty acid synthesis	SCD	hsa-let-7f-5p	-0.36
de novo fatty acid synthesis	SCD	hsa-let-7g-5p	-0.35
de novo fatty acid synthesis	SCD	hsa-let-7i-5p	-0.35
de novo fatty acid synthesis	SCD	hsa-miR-124-3p.1	-0.22
de novo fatty acid synthesis	SCD	hsa-miR-124-3p.2	-0.13
de novo fatty acid synthesis	SCD	hsa-miR-125a-5p	-0.15
de novo fatty acid synthesis	SCD	hsa-miR-125b-5p	-0.15
de novo fatty acid synthesis	SCD	hsa-miR-128-3p	-0.05

de novo fatty acid synthesis	SCD	hsa-miR-1-3p	-0.12
de novo fatty acid synthesis	SCD	hsa-miR-181a-5p	-0.31
de novo fatty acid synthesis	SCD	hsa-miR-181b-5p	-0.31
de novo fatty acid synthesis	SCD	hsa-miR-181c-5p	-0.31
de novo fatty acid synthesis	SCD	hsa-miR-181d-5p	-0.31
de novo fatty acid synthesis	SCD	hsa-miR-186-5p	-0.05
de novo fatty acid synthesis	SCD	hsa-miR-199a-3p	-0.57
de novo fatty acid synthesis	SCD	hsa-miR-199b-3p	-0.57
de novo fatty acid synthesis	SCD	hsa-miR-200b-3p	-0.26
de novo fatty acid synthesis	SCD	hsa-miR-200c-3p	-0.26
de novo fatty acid synthesis	SCD	hsa-miR-206	-0.12
de novo fatty acid synthesis	SCD	hsa-miR-216a-3p	-0.06
de novo fatty acid synthesis	SCD	hsa-miR-3129-5p	-0.48
de novo fatty acid synthesis	SCD	hsa-miR-3681-3p	-0.02
de novo fatty acid synthesis	SCD	hsa-miR-370-5p	-0.03
de novo fatty acid synthesis	SCD	hsa-miR-382-5p	-0.03
de novo fatty acid synthesis	SCD	hsa-miR-4262	-0.22
de novo fatty acid synthesis	SCD	hsa-miR-429	-0.25
de novo fatty acid synthesis	SCD	hsa-miR-4319	-0.13
de novo fatty acid synthesis	SCD	hsa-miR-4458	-0.35
de novo fatty acid synthesis	SCD	hsa-miR-4500	-0.34
de novo fatty acid synthesis	SCD	hsa-miR-495-3p	-0.03
de novo fatty acid synthesis	SCD	hsa-miR-506-3p	-0.08
de novo fatty acid synthesis	SCD	hsa-miR-5688	-0.02
de novo fatty acid synthesis	SCD	hsa-miR-613	-0.13
de novo fatty acid synthesis	SCD	hsa-miR-98-5p	-0.37
Glutaminolysis	GLS1	hsa-miR-1-3p	-0.29
Glutaminolysis	GLS1	hsa-miR-206	-0.28
Glutaminolysis	GLS1	hsa-miR-300	-0.01
Glutaminolysis	GLS1	hsa-miR-330-3p	-0.11
Glutaminolysis	GLS1	hsa-miR-381-3p	-0.03
Glutaminolysis	GLS1	hsa-miR-613	-0.27

Glutaminolysis	GLS2	hsa-miR-15a-5p	-0.32
Glutaminolysis	GLS2	hsa-miR-15b-5p	-0.33
Glutaminolysis	GLS2	hsa-miR-16-5p	-0.31
Glutaminolysis	GLS2	hsa-miR-195-5p	-0.31
Glutaminolysis	GLS2	hsa-miR-424-5p	-0.37
Glutaminolysis	GLS2	hsa-miR-497-5p	-0.33
Glutaminolysis	GLS2	hsa-miR-6838-5p	-0.32
PPP pathway	G6PD	hsa-miR-133a-3p.1	-0.33
PPP pathway	G6PD	hsa-miR-1-3p	-0.26
PPP pathway	G6PD	hsa-miR-206	-0.27
PPP pathway	G6PD	hsa-miR-24-3p	-0.2
PPP pathway	G6PD	hsa-miR-613	-0.21
PPP pathway	TKTL1	hsa-miR-203a-3p.1	-0.23
Serine, glycine and one carbon metabolism	MTHFD1L	hsa-miR-103a-3p	-0.27
Serine, glycine and one carbon metabolism	MTHFD1L	hsa-miR-107	-0.27
Serine, glycine and one carbon metabolism	MTHFD1L	hsa-miR-296-3p	-0.12
Serine, glycine and one carbon metabolism	MTHFD1L	hsa-miR-338-3p	-0.42
Serine, glycine and one carbon metabolism	MTHFD1L	hsa-miR-362-5p	-0.08
Serine, glycine and one carbon metabolism	MTHFD1L	hsa-miR-377-3p	-0.2
Serine, glycine and one carbon metabolism	MTHFD1L	hsa-miR-455-5p	-0.15
Serine, glycine and one carbon metabolism	MTHFD1L	hsa-miR-500b-5p	-0.1
Serine, glycine and one carbon metabolism	MTHFD1L	hsa-miR-543	-0.19
Serine, glycine and one carbon metabolism	MTHFD1L	hsa-miR-873-5p.1	-0.03
Serine, glycine and one carbon metabolism	MTHFD1L	hsa-miR-873-5p.2	-0.03
Serine, glycine and one carbon metabolism	MTHFD1L	hsa-miR-877-5p	-0.02
Serine, glycine and one carbon metabolism	MTHFD1L	hsa-miR-9-5p	-0.18
Serine, glycine and one carbon metabolism	MTHFD2	hsa-miR-1251-5p	-0.5
Serine, glycine and one carbon metabolism	MTHFD2	hsa-miR-134-5p	-0.03
Serine, glycine and one carbon metabolism	MTHFD2	hsa-miR-22-3p	-0.33
Serine, glycine and one carbon metabolism	MTHFD2	hsa-miR-25-3p	-0.22
Serine, glycine and one carbon metabolism	MTHFD2	hsa-miR-3064-5p	-0.19
Serine, glycine and one carbon metabolism	MTHFD2	hsa-miR-3118	-0.03

Serine, glycine and one carbon metabolism	MTHFD2	hsa-miR-32-5p	-0.22
Serine, glycine and one carbon metabolism	MTHFD2	hsa-miR-33a-5p	-0.15
Serine, glycine and one carbon metabolism	MTHFD2	hsa-miR-33b-5p	-0.15
Serine, glycine and one carbon metabolism	MTHFD2	hsa-miR-363-3p	-0.22
Serine, glycine and one carbon metabolism	MTHFD2	hsa-miR-367-3p	-0.2
Serine, glycine and one carbon metabolism	MTHFD2	hsa-miR-6504-5p	-0.19
Serine, glycine and one carbon metabolism	MTHFD2	hsa-miR-92a-3p	-0.23
Serine, glycine and one carbon metabolism	MTHFD2	hsa-miR-92b-3p	-0.22
Serine, glycine and one carbon metabolism	MTHFD2	hsa-miR-9-5p	-0.31
Serine, glycine and one carbon metabolism	PHGDH	hsa-miR-128-3p	-0.44
Serine, glycine and one carbon metabolism	PHGDH	hsa-miR-216a-3p	-0.44
Serine, glycine and one carbon metabolism	PHGDH	hsa-miR-3681-3p	-0.43
Serine, glycine and one carbon metabolism	PSAT1	hsa-miR-145-5p	-0.36
Serine, glycine and one carbon metabolism	PSAT1	hsa-miR-15a-5p	-0.38
Serine, glycine and one carbon metabolism	PSAT1	hsa-miR-15b-5p	-0.38
Serine, glycine and one carbon metabolism	PSAT1	hsa-miR-16-5p	-0.42
Serine, glycine and one carbon metabolism	PSAT1	hsa-miR-195-5p	-0.42
Serine, glycine and one carbon metabolism	PSAT1	hsa-miR-200b-3p	-0.26
Serine, glycine and one carbon metabolism	PSAT1	hsa-miR-200c-3p	-0.26
Serine, glycine and one carbon metabolism	PSAT1	hsa-miR-424-5p	-0.4
Serine, glycine and one carbon metabolism	PSAT1	hsa-miR-429	-0.27
Serine, glycine and one carbon metabolism	PSAT1	hsa-miR-497-5p	-0.39
Serine, glycine and one carbon metabolism	PSAT1	hsa-miR-5195-3p	-0.42
Serine, glycine and one carbon metabolism	PSAT1	hsa-miR-6838-5p	-0.44
Serine, glycine and one carbon metabolism	PSPH	hsa-miR-139-5p	-0.31
Serine, glycine and one carbon metabolism	PSPH	hsa-miR-186-5p	-0.2
Serine, glycine and one carbon metabolism	PSPH	hsa-miR-200b-3p	-0.28
Serine, glycine and one carbon metabolism	PSPH	hsa-miR-200c-3p	-0.28
Serine, glycine and one carbon metabolism	PSPH	hsa-miR-330-3p	-0.19
Serine, glycine and one carbon metabolism	PSPH	hsa-miR-382-5p	-0.21
Serine, glycine and one carbon metabolism	PSPH	hsa-miR-429	-0.29
Serine, glycine and one carbon metabolism	SHMT1	hsa-miR-149-5p	-0.01

Serine, glycine and one carbon metabolism	SHMT1	hsa-miR-217	-0.16
Serine, glycine and one carbon metabolism	SHMT1	hsa-miR-218-5p	-0.56
Serine, glycine and one carbon metabolism	SHMT1	hsa-miR-3064-5p	-0.09
Serine, glycine and one carbon metabolism	SHMT1	hsa-miR-320a	-0.17
Serine, glycine and one carbon metabolism	SHMT1	hsa-miR-320b	-0.17
Serine, glycine and one carbon metabolism	SHMT1	hsa-miR-320c	-0.17
Serine, glycine and one carbon metabolism	SHMT1	hsa-miR-320d	-0.17
Serine, glycine and one carbon metabolism	SHMT1	hsa-miR-4429	-0.17
Serine, glycine and one carbon metabolism	SHMT1	hsa-miR-6504-5p	-0.1
Serine, glycine and one carbon metabolism	SHMT1	hsa-miR-6807-3p	-0.21
Serine, glycine and one carbon metabolism	SHMT1	hsa-miR-9-5p	-0.1
Serine, glycine and one carbon metabolism	SHMT2	hsa-miR-149-5p	-0.32
Serine, glycine and one carbon metabolism	SHMT2	hsa-miR-383-5p.1	-0.31
Serine, glycine and one carbon metabolism	SHMT2	hsa-miR-485-5p	-0.04
Serine, glycine and one carbon metabolism	SHMT2	hsa-miR-495-3p	-0.06
Serine, glycine and one carbon metabolism	SHMT2	hsa-miR-5688	-0.01
Serine, glycine and one carbon metabolism	SHMT2	hsa-miR-6884-5p	-0.04
Serine, glycine and one carbon metabolism	SHMT2	hsa-miR-760	-0.16
TCA cycle	IDH1	hsa-miR-130a-5p	-0.27
TCA cycle	IDH1	hsa-miR-133a-3p.2	-0.25
TCA cycle	IDH1	hsa-miR-133b	-0.25
TCA cycle	IDH1	hsa-miR-137	-0.22
TCA cycle	IDH1	hsa-miR-142-3p.2	-0.3
TCA cycle	IDH1	hsa-miR-23a-3p	-0.25
TCA cycle	IDH1	hsa-miR-23b-3p	-0.25
TCA cycle	IDH1	hsa-miR-23c	-0.25
TCA cycle	IDH1	hsa-miR-25-3p	-0.55
TCA cycle	IDH1	hsa-miR-30a-5p	-0.28
TCA cycle	IDH1	hsa-miR-30b-5p	-0.25
TCA cycle	IDH1	hsa-miR-30c-5p	-0.25
TCA cycle	IDH1	hsa-miR-30d-5p	-0.28
TCA cycle	IDH1	hsa-miR-30e-5p	-0.28

TCA cycle	IDH1	hsa-miR-32-5p	-0.54
TCA cycle	IDH1	hsa-miR-363-3p	-0.55
TCA cycle	IDH1	hsa-miR-367-3p	-0.56
TCA cycle	IDH1	hsa-miR-92a-3p	-0.53
TCA cycle	IDH1	hsa-miR-92b-3p	-0.5
TCA cycle	IDH2	hsa-let-7a-5p	-0.31
TCA cycle	IDH2	hsa-let-7b-5p	-0.31
TCA cycle	IDH2	hsa-let-7c-5p	-0.31
TCA cycle	IDH2	hsa-let-7d-5p	-0.36
TCA cycle	IDH2	hsa-let-7e-5p	-0.31
TCA cycle	IDH2	hsa-let-7f-5p	-0.31
TCA cycle	IDH2	hsa-let-7g-5p	-0.31
TCA cycle	IDH2	hsa-let-7i-5p	-0.32
TCA cycle	IDH2	hsa-miR-101-3p.1	-0.18
TCA cycle	IDH2	hsa-miR-101-3p.2	-0.38
TCA cycle	IDH2	hsa-miR-144-3p	-0.4
TCA cycle	IDH2	hsa-miR-183-5p.1	-0.67
TCA cycle	IDH2	hsa-miR-3064-5p	-0.38
TCA cycle	IDH2	hsa-miR-3184-5p	-0.19
TCA cycle	IDH2	hsa-miR-340-5p	-0.01
TCA cycle	IDH2	hsa-miR-411-5p.2	-0.19
TCA cycle	IDH2	hsa-miR-423-5p	-0.17
TCA cycle	IDH2	hsa-miR-4458	-0.3
TCA cycle	IDH2	hsa-miR-4500	-0.31
TCA cycle	IDH2	hsa-miR-582-5p	-0.17
TCA cycle	IDH2	hsa-miR-6504-5p	-0.41
TCA cycle	IDH2	hsa-miR-758-3p	-0.05
TCA cycle	IDH2	hsa-miR-98-5p	-0.31
TCA cycle	ME1	hsa-miR-153-3p	-0.46
TCA cycle	ME1	hsa-miR-30a-5p	-0.03
TCA cycle	ME1	hsa-miR-30b-5p	-0.03
TCA cycle	ME1	hsa-miR-30c-5p	-0.03

TCA cycle	ME1	hsa-miR-30d-5p	-0.03
TCA cycle	ME1	hsa-miR-30e-5p	-0.03
TCA cycle	ME1	hsa-miR-448	-0.07
TCA cycle	PDHX	hsa-miR-128-3p	-0.45
TCA cycle	PDHX	hsa-miR-135a-5p	-0.44
TCA cycle	PDHX	hsa-miR-135b-5p	-0.43
TCA cycle	PDHX	hsa-miR-154-3p	-0.35
TCA cycle	PDHX	hsa-miR-181a-5p	-0.19
TCA cycle	PDHX	hsa-miR-181b-5p	-0.19
TCA cycle	PDHX	hsa-miR-181c-5p	-0.19
TCA cycle	PDHX	hsa-miR-181d-5p	-0.19
TCA cycle	PDHX	hsa-miR-216a-3p	-0.45
TCA cycle	PDHX	hsa-miR-27a-3p	-0.38
TCA cycle	PDHX	hsa-miR-27b-3p	-0.38
TCA cycle	PDHX	hsa-miR-29a-3p	-0.31
TCA cycle	PDHX	hsa-miR-29b-3p	-0.31
TCA cycle	PDHX	hsa-miR-29c-3p	-0.31
TCA cycle	PDHX	hsa-miR-302c-3p.2	-0.19
TCA cycle	PDHX	hsa-miR-3681-3p	-0.48
TCA cycle	PDHX	hsa-miR-409-3p	-0.06
TCA cycle	PDHX	hsa-miR-4262	-0.12
TCA cycle	PDHX	hsa-miR-487a-3p	-0.35
TCA cycle	PDHX	hsa-miR-520f-3p	-0.21
TCA cycle	PDK1	hsa-miR-1271-5p	-0.25
TCA cycle	PDK1	hsa-miR-128-3p	-0.21
TCA cycle	PDK1	hsa-miR-138-5p	-0.39
TCA cycle	PDK1	hsa-miR-216a-3p	-0.2
TCA cycle	PDK1	hsa-miR-27a-3p	-0.26
TCA cycle	PDK1	hsa-miR-27b-3p	-0.26
TCA cycle	PDK1	hsa-miR-3681-3p	-0.21
TCA cycle	PDK1	hsa-miR-409-3p	-0.02
TCA cycle	PDK1	hsa-miR-96-5p	-0.29

TCA cycle	SDHC	hsa-miR-1251-5p	-0.19
TCA cycle	SDHC	hsa-miR-140-3p.1	-0.13
TCA cycle	SDHC	hsa-miR-185-5p	-0.43
TCA cycle	SDHC	hsa-miR-216b-5p	-0.11
TCA cycle	SDHC	hsa-miR-320a	-0.09
TCA cycle	SDHC	hsa-miR-320b	-0.09
TCA cycle	SDHC	hsa-miR-320c	-0.09
TCA cycle	SDHC	hsa-miR-320d	-0.09
TCA cycle	SDHC	hsa-miR-34a-5p	-0.21
TCA cycle	SDHC	hsa-miR-34c-5p	-0.21
TCA cycle	SDHC	hsa-miR-365a-3p	-0.45
TCA cycle	SDHC	hsa-miR-365b-3p	-0.45
TCA cycle	SDHC	hsa-miR-4306	-0.43
TCA cycle	SDHC	hsa-miR-4429	-0.09
TCA cycle	SDHC	hsa-miR-449a	-0.21
TCA cycle	SDHC	hsa-miR-449b-5p	-0.2
TCA cycle	SDHC	hsa-miR-4644	-0.43
TCA cycle	SDHC	hsa-miR-496.1	-0.62
TCA cycle	SDHD	hsa-miR-130a-5p	-0.37
TCA cycle	SDHD	hsa-miR-140-3p.1	-0.16
TCA cycle	SDHD	hsa-miR-204-5p	-0.35
TCA cycle	SDHD	hsa-miR-211-5p	-0.35
TCA cycle	SDHD	hsa-miR-212-5p	-0.14
TCA cycle	SDHD	hsa-miR-23a-3p	-0.42
TCA cycle	SDHD	hsa-miR-23b-3p	-0.42
TCA cycle	SDHD	hsa-miR-23c	-0.42
TCA cycle	SDHD	hsa-miR-320a	-0.35
TCA cycle	SDHD	hsa-miR-320b	-0.35
TCA cycle	SDHD	hsa-miR-320c	-0.35
TCA cycle	SDHD	hsa-miR-320d	-0.35
TCA cycle	SDHD	hsa-miR-4429	-0.35
