# Supplementary material for: MicroRNAs and oncogenic transcriptional regulatory networks controlling metabolic reprogramming in cancers
Source: Comput Struct Biotechnol J. 2016 Jun 4;14:223–33. doi: 10.1016/j.csbj.2016.05.005 (PMC4915959; doi:10.1016/j.csbj.2016.05.005)
Supplement: Table S2 — Prediction of miRNAs that regulate metabolic enzymes by miRanda–mirSVR. [file mmc2.pdf]

Table S2

Metabolic pathways	Target genes	miRNA family	mirSVR scores
Anaerobic glycolysis	ALDOA	hsa-miR-1271	-0.1234
Anaerobic glycolysis	ALDOA	hsa-miR-182	-0.1222
Anaerobic glycolysis	ALDOA	hsa-miR-329	-0.3496
Anaerobic glycolysis	ALDOA	hsa-miR-34a	-1.0804
Anaerobic glycolysis	ALDOA	hsa-miR-34c-5p	-1.0829
Anaerobic glycolysis	ALDOA	hsa-miR-362-3p	-0.3468
Anaerobic glycolysis	ALDOA	hsa-miR-449a	-1.0754
Anaerobic glycolysis	ALDOA	hsa-miR-449b	-1.0754
Anaerobic glycolysis	ALDOA	hsa-miR-96	-0.1259
Anaerobic glycolysis	GLUT1	hsa-miR-130a	-0.2414
Anaerobic glycolysis	GLUT1	hsa-miR-130b	-0.2393
Anaerobic glycolysis	GLUT1	hsa-miR-132	-0.7526
Anaerobic glycolysis	GLUT1	hsa-miR-138	-0.1478
Anaerobic glycolysis	GLUT1	hsa-miR-140-5p	-0.5577
Anaerobic glycolysis	GLUT1	hsa-miR-143	-0.4377
Anaerobic glycolysis	GLUT1	hsa-miR-144	-0.909
Anaerobic glycolysis	GLUT1	hsa-miR-148a	-0.6927
Anaerobic glycolysis	GLUT1	hsa-miR-148b	-0.6927
Anaerobic glycolysis	GLUT1	hsa-miR-150	-0.146
Anaerobic glycolysis	GLUT1	hsa-miR-152	-0.6927
Anaerobic glycolysis	GLUT1	hsa-miR-181a	-0.7512
Anaerobic glycolysis	GLUT1	hsa-miR-181b	-0.7512
Anaerobic glycolysis	GLUT1	hsa-miR-181c	-0.7512
Anaerobic glycolysis	GLUT1	hsa-miR-181d	-0.7512
Anaerobic glycolysis	GLUT1	hsa-miR-193a-3p	-0.1522
Anaerobic glycolysis	GLUT1	hsa-miR-193b	-0.1522
Anaerobic glycolysis	GLUT1	hsa-miR-194	-0.6482
Anaerobic glycolysis	GLUT1	hsa-miR-19a	-0.1236
Anaerobic glycolysis	GLUT1	hsa-miR-19b	-0.1236

Anaerobic glycolysis	GLUT1	hsa-miR-203	-0.2808
Anaerobic glycolysis	GLUT1	hsa-miR-21	-0.7251
Anaerobic glycolysis	GLUT1	hsa-miR-212	-0.7636
Anaerobic glycolysis	GLUT1	hsa-miR-22	-0.1895
Anaerobic glycolysis	GLUT1	hsa-miR-23a	-0.6856
Anaerobic glycolysis	GLUT1	hsa-miR-23b	-0.6856
Anaerobic glycolysis	GLUT1	hsa-miR-299-3p	-0.2039
Anaerobic glycolysis	GLUT1	hsa-miR-301a	-0.2272
Anaerobic glycolysis	GLUT1	hsa-miR-301b	-0.2436
Anaerobic glycolysis	GLUT1	hsa-miR-302a	-0.1213
Anaerobic glycolysis	GLUT1	hsa-miR-302b	-0.1213
Anaerobic glycolysis	GLUT1	hsa-miR-302c	-0.1213
Anaerobic glycolysis	GLUT1	hsa-miR-302d	-0.1213
Anaerobic glycolysis	GLUT1	hsa-miR-302e	-0.1225
Anaerobic glycolysis	GLUT1	hsa-miR-326	-1.1603
Anaerobic glycolysis	GLUT1	hsa-miR-328	-0.421
Anaerobic glycolysis	GLUT1	hsa-miR-330-5p	-1.1603
Anaerobic glycolysis	GLUT1	hsa-miR-340	-0.1724
Anaerobic glycolysis	GLUT1	hsa-miR-370	-0.2574
Anaerobic glycolysis	GLUT1	hsa-miR-372	-0.1189
Anaerobic glycolysis	GLUT1	hsa-miR-373	-0.1213
Anaerobic glycolysis	GLUT1	hsa-miR-378	-0.6517
Anaerobic glycolysis	GLUT1	hsa-miR-410	-0.2913
Anaerobic glycolysis	GLUT1	hsa-miR-422a	-0.6858
Anaerobic glycolysis	GLUT1	hsa-miR-431	-0.9929
Anaerobic glycolysis	GLUT1	hsa-miR-454	-0.2414
Anaerobic glycolysis	GLUT1	hsa-miR-490-3p	-0.2242
Anaerobic glycolysis	GLUT1	hsa-miR-491-5p	-0.2101
Anaerobic glycolysis	GLUT1	hsa-miR-495	-0.4829
Anaerobic glycolysis	GLUT1	hsa-miR-520a-3p	-0.1237
Anaerobic glycolysis	GLUT1	hsa-miR-520b	-0.1225
Anaerobic glycolysis	GLUT1	hsa-miR-520c-3p	-0.1225

Anaerobic glycolysis	GLUT1	hsa-miR-520d-3p	-0.1237
Anaerobic glycolysis	GLUT1	hsa-miR-520e	-0.1225
Anaerobic glycolysis	GLUT1	hsa-miR-590-5p	-0.7436
Anaerobic glycolysis	GLUT1	hsa-miR-873	-0.1135
Anaerobic glycolysis	GLUT1	hsa-miR-9	-0.6817
Anaerobic glycolysis	GLUT2	hsa-let-7a	-0.1535
Anaerobic glycolysis	GLUT2	hsa-let-7b	-0.1535
Anaerobic glycolysis	GLUT2	hsa-let-7c	-0.1535
Anaerobic glycolysis	GLUT2	hsa-let-7d	-0.1505
Anaerobic glycolysis	GLUT2	hsa-let-7e	-0.1535
Anaerobic glycolysis	GLUT2	hsa-let-7f	-0.152
Anaerobic glycolysis	GLUT2	hsa-let-7g	-0.1535
Anaerobic glycolysis	GLUT2	hsa-let-7i	-0.1535
Anaerobic glycolysis	GLUT2	hsa-miR-106a	-0.9436
Anaerobic glycolysis	GLUT2	hsa-miR-106b	-0.9436
Anaerobic glycolysis	GLUT2	hsa-miR-125a-3p	-1.0497
Anaerobic glycolysis	GLUT2	hsa-miR-129-5p	-0.2522
Anaerobic glycolysis	GLUT2	hsa-miR-130a	-0.6215
Anaerobic glycolysis	GLUT2	hsa-miR-130b	-0.6215
Anaerobic glycolysis	GLUT2	hsa-miR-140-5p	-0.9978
Anaerobic glycolysis	GLUT2	hsa-miR-17	-0.9436
Anaerobic glycolysis	GLUT2	hsa-miR-190	-1.1088
Anaerobic glycolysis	GLUT2	hsa-miR-190b	-1.111
Anaerobic glycolysis	GLUT2	hsa-miR-200b	-0.1234
Anaerobic glycolysis	GLUT2	hsa-miR-200c	-0.1234
Anaerobic glycolysis	GLUT2	hsa-miR-202	-0.152
Anaerobic glycolysis	GLUT2	hsa-miR-20a	-0.9404
Anaerobic glycolysis	GLUT2	hsa-miR-20b	-0.9404
Anaerobic glycolysis	GLUT2	hsa-miR-216b	-0.7882
Anaerobic glycolysis	GLUT2	hsa-miR-219-5p	-0.662
Anaerobic glycolysis	GLUT2	hsa-miR-299-3p	-0.1143
Anaerobic glycolysis	GLUT2	hsa-miR-301a	-0.6289

Anaerobic glycolysis	GLUT2	hsa-miR-301b	-0.6289
Anaerobic glycolysis	GLUT2	hsa-miR-302a	-0.9025
Anaerobic glycolysis	GLUT2	hsa-miR-302b	-0.9025
Anaerobic glycolysis	GLUT2	hsa-miR-302c	-0.9025
Anaerobic glycolysis	GLUT2	hsa-miR-302d	-0.9025
Anaerobic glycolysis	GLUT2	hsa-miR-302e	-0.8991
Anaerobic glycolysis	GLUT2	hsa-miR-320a	-0.6293
Anaerobic glycolysis	GLUT2	hsa-miR-320b	-0.6293
Anaerobic glycolysis	GLUT2	hsa-miR-320c	-0.6293
Anaerobic glycolysis	GLUT2	hsa-miR-320d	-0.6293
Anaerobic glycolysis	GLUT2	hsa-miR-329	-0.6021
Anaerobic glycolysis	GLUT2	hsa-miR-340	-0.898
Anaerobic glycolysis	GLUT2	hsa-miR-362-3p	-0.6057
Anaerobic glycolysis	GLUT2	hsa-miR-371-5p	-0.1547
Anaerobic glycolysis	GLUT2	hsa-miR-372	-0.9092
Anaerobic glycolysis	GLUT2	hsa-miR-373	-0.9025
Anaerobic glycolysis	GLUT2	hsa-miR-374a	-0.8355
Anaerobic glycolysis	GLUT2	hsa-miR-374b	-0.8425
Anaerobic glycolysis	GLUT2	hsa-miR-410	-0.2046
Anaerobic glycolysis	GLUT2	hsa-miR-425	-0.1122
Anaerobic glycolysis	GLUT2	hsa-miR-429	-0.1246
Anaerobic glycolysis	GLUT2	hsa-miR-433	-0.2157
Anaerobic glycolysis	GLUT2	hsa-miR-454	-0.64
Anaerobic glycolysis	GLUT2	hsa-miR-495	-0.1673
Anaerobic glycolysis	GLUT2	hsa-miR-519d	-0.9307
Anaerobic glycolysis	GLUT2	hsa-miR-520a-3p	-0.9058
Anaerobic glycolysis	GLUT2	hsa-miR-520b	-0.9025
Anaerobic glycolysis	GLUT2	hsa-miR-520c-3p	-0.9025
Anaerobic glycolysis	GLUT2	hsa-miR-520d-3p	-0.9058
Anaerobic glycolysis	GLUT2	hsa-miR-520e	-0.8991
Anaerobic glycolysis	GLUT2	hsa-miR-590-3p	-0.8719
Anaerobic glycolysis	GLUT2	hsa-miR-653	-0.7855

Anaerobic glycolysis	GLUT2	hsa-miR-93	-0.9372
Anaerobic glycolysis	GLUT2	hsa-miR-98	-0.1535
Anaerobic glycolysis	GLUT3	hsa-miR-103	-0.539
Anaerobic glycolysis	GLUT3	hsa-miR-107	-0.539
Anaerobic glycolysis	GLUT3	hsa-miR-1271	-0.2232
Anaerobic glycolysis	GLUT3	hsa-miR-128	-0.1534
Anaerobic glycolysis	GLUT3	hsa-miR-129-5p	-0.5213
Anaerobic glycolysis	GLUT3	hsa-miR-1297	-0.7952
Anaerobic glycolysis	GLUT3	hsa-miR-146a	-0.5799
Anaerobic glycolysis	GLUT3	hsa-miR-146b-5p	-0.5799
Anaerobic glycolysis	GLUT3	hsa-miR-148a	-0.2064
Anaerobic glycolysis	GLUT3	hsa-miR-148b	-0.192
Anaerobic glycolysis	GLUT3	hsa-miR-152	-0.2064
Anaerobic glycolysis	GLUT3	hsa-miR-15a	-0.9241
Anaerobic glycolysis	GLUT3	hsa-miR-15b	-0.9273
Anaerobic glycolysis	GLUT3	hsa-miR-16	-0.9306
Anaerobic glycolysis	GLUT3	hsa-miR-181a	-0.7697
Anaerobic glycolysis	GLUT3	hsa-miR-181b	-0.7697
Anaerobic glycolysis	GLUT3	hsa-miR-181c	-0.7733
Anaerobic glycolysis	GLUT3	hsa-miR-181d	-0.766
Anaerobic glycolysis	GLUT3	hsa-miR-182	-1.2387
Anaerobic glycolysis	GLUT3	hsa-miR-18a	-0.1483
Anaerobic glycolysis	GLUT3	hsa-miR-18b	-0.1483
Anaerobic glycolysis	GLUT3	hsa-miR-194	-0.2556
Anaerobic glycolysis	GLUT3	hsa-miR-195	-0.9306
Anaerobic glycolysis	GLUT3	hsa-miR-200b	-0.2304
Anaerobic glycolysis	GLUT3	hsa-miR-200c	-0.2304
Anaerobic glycolysis	GLUT3	hsa-miR-203	-0.1324
Anaerobic glycolysis	GLUT3	hsa-miR-216a	-1.0841
Anaerobic glycolysis	GLUT3	hsa-miR-25	-0.7688
Anaerobic glycolysis	GLUT3	hsa-miR-26a	-0.8116
Anaerobic glycolysis	GLUT3	hsa-miR-26b	-0.7989

Anaerobic glycolysis	GLUT3	hsa-miR-29a	-0.3123
Anaerobic glycolysis	GLUT3	hsa-miR-29b	-0.3123
Anaerobic glycolysis	GLUT3	hsa-miR-29c	-0.3123
Anaerobic glycolysis	GLUT3	hsa-miR-301a	-0.3171
Anaerobic glycolysis	GLUT3	hsa-miR-32	-0.7761
Anaerobic glycolysis	GLUT3	hsa-miR-320a	-0.1441
Anaerobic glycolysis	GLUT3	hsa-miR-320b	-0.1441
Anaerobic glycolysis	GLUT3	hsa-miR-320c	-0.1441
Anaerobic glycolysis	GLUT3	hsa-miR-320d	-0.1441
Anaerobic glycolysis	GLUT3	hsa-miR-338-3p	-0.7322
Anaerobic glycolysis	GLUT3	hsa-miR-361-5p	-0.1243
Anaerobic glycolysis	GLUT3	hsa-miR-363	-0.7675
Anaerobic glycolysis	GLUT3	hsa-miR-365	-0.7026
Anaerobic glycolysis	GLUT3	hsa-miR-367	-0.7761
Anaerobic glycolysis	GLUT3	hsa-miR-424	-0.9051
Anaerobic glycolysis	GLUT3	hsa-miR-429	-0.2284
Anaerobic glycolysis	GLUT3	hsa-miR-455-5p	-0.2362
Anaerobic glycolysis	GLUT3	hsa-miR-490-3p	-0.6992
Anaerobic glycolysis	GLUT3	hsa-miR-495	-0.316
Anaerobic glycolysis	GLUT3	hsa-miR-497	-0.9306
Anaerobic glycolysis	GLUT3	hsa-miR-505	-0.6883
Anaerobic glycolysis	GLUT3	hsa-miR-539	-0.1065
Anaerobic glycolysis	GLUT3	hsa-miR-542-3p	-0.3196
Anaerobic glycolysis	GLUT3	hsa-miR-543	-0.6881
Anaerobic glycolysis	GLUT3	hsa-miR-92a	-0.7761
Anaerobic glycolysis	GLUT3	hsa-miR-92b	-0.7761
Anaerobic glycolysis	GLUT3	hsa-miR-96	-0.2232
Anaerobic glycolysis	GLUT4	hsa-miR-106a	-0.9689
Anaerobic glycolysis	GLUT4	hsa-miR-106b	-0.9689
Anaerobic glycolysis	GLUT4	hsa-miR-16	-0.3145
Anaerobic glycolysis	GLUT4	hsa-miR-17	-0.9689
Anaerobic glycolysis	GLUT4	hsa-miR-183	-0.1276

Anaerobic glycolysis	GLUT4	hsa-miR-199a-5p	-0.2614
Anaerobic glycolysis	GLUT4	hsa-miR-199b-5p	-0.2614
Anaerobic glycolysis	GLUT4	hsa-miR-20a	-0.972
Anaerobic glycolysis	GLUT4	hsa-miR-20b	-0.972
Anaerobic glycolysis	GLUT4	hsa-miR-302a	-0.4508
Anaerobic glycolysis	GLUT4	hsa-miR-302b	-0.4508
Anaerobic glycolysis	GLUT4	hsa-miR-302c	-0.4508
Anaerobic glycolysis	GLUT4	hsa-miR-302d	-0.4508
Anaerobic glycolysis	GLUT4	hsa-miR-302e	-0.4508
Anaerobic glycolysis	GLUT4	hsa-miR-31	-0.7789
Anaerobic glycolysis	GLUT4	hsa-miR-335	-0.1542
Anaerobic glycolysis	GLUT4	hsa-miR-339-5p	-0.3838
Anaerobic glycolysis	GLUT4	hsa-miR-372	-0.4475
Anaerobic glycolysis	GLUT4	hsa-miR-373	-0.4574
Anaerobic glycolysis	GLUT4	hsa-miR-491-5p	-0.1486
Anaerobic glycolysis	GLUT4	hsa-miR-503	-0.5094
Anaerobic glycolysis	GLUT4	hsa-miR-519d	-0.9841
Anaerobic glycolysis	GLUT4	hsa-miR-520a-3p	-0.4475
Anaerobic glycolysis	GLUT4	hsa-miR-520b	-0.4508
Anaerobic glycolysis	GLUT4	hsa-miR-520c-3p	-0.4508
Anaerobic glycolysis	GLUT4	hsa-miR-520d-3p	-0.4475
Anaerobic glycolysis	GLUT4	hsa-miR-520e	-0.4574
Anaerobic glycolysis	GLUT4	hsa-miR-874	-0.1711
Anaerobic glycolysis	GLUT4	hsa-miR-876-5p	-0.3509
Anaerobic glycolysis	GLUT4	hsa-miR-9	-0.729
Anaerobic glycolysis	GLUT4	hsa-miR-93	-0.9781
Anaerobic glycolysis	HK1	hsa-miR-138	-0.7056
Anaerobic glycolysis	HK1	hsa-miR-145	-0.1003
Anaerobic glycolysis	HK1	hsa-miR-155	-0.168
Anaerobic glycolysis	HK1	hsa-miR-302a	-0.1045
Anaerobic glycolysis	HK1	hsa-miR-302b	-0.1045
Anaerobic glycolysis	HK1	hsa-miR-302c	-0.1045

Anaerobic glycolysis	HK1	hsa-miR-302d	-0.1045
Anaerobic glycolysis	HK1	hsa-miR-302e	-0.1024
Anaerobic glycolysis	HK1	hsa-miR-329	-0.2101
Anaerobic glycolysis	HK1	hsa-miR-362-3p	-0.2063
Anaerobic glycolysis	HK1	hsa-miR-372	-0.1045
Anaerobic glycolysis	HK1	hsa-miR-373	-0.1056
Anaerobic glycolysis	HK1	hsa-miR-377	-0.416
Anaerobic glycolysis	HK1	hsa-miR-421	-0.6353
Anaerobic glycolysis	HK1	hsa-miR-424	-0.2067
Anaerobic glycolysis	HK1	hsa-miR-520a-3p	-0.1045
Anaerobic glycolysis	HK1	hsa-miR-520b	-0.1045
Anaerobic glycolysis	HK1	hsa-miR-520c-3p	-0.1045
Anaerobic glycolysis	HK1	hsa-miR-520d-3p	-0.1045
Anaerobic glycolysis	HK1	hsa-miR-520e	-0.1066
Anaerobic glycolysis	HK2	hsa-miR-1	-0.7014
Anaerobic glycolysis	HK2	hsa-miR-125a-5p	-0.6958
Anaerobic glycolysis	HK2	hsa-miR-125b	-0.707
Anaerobic glycolysis	HK2	hsa-miR-143	-0.8122
Anaerobic glycolysis	HK2	hsa-miR-146b-5p	-0.7017
Anaerobic glycolysis	HK2	hsa-miR-150	-0.2528
Anaerobic glycolysis	HK2	hsa-miR-181a	-0.8986
Anaerobic glycolysis	HK2	hsa-miR-181b	-0.8986
Anaerobic glycolysis	HK2	hsa-miR-181c	-0.902
Anaerobic glycolysis	HK2	hsa-miR-181d	-0.8953
Anaerobic glycolysis	HK2	hsa-miR-182	-0.3259
Anaerobic glycolysis	HK2	hsa-miR-185	-0.3904
Anaerobic glycolysis	HK2	hsa-miR-199a-5p	-0.8668
Anaerobic glycolysis	HK2	hsa-miR-199b-5p	-0.8633
Anaerobic glycolysis	HK2	hsa-miR-200b	-0.1039
Anaerobic glycolysis	HK2	hsa-miR-200c	-0.1039
Anaerobic glycolysis	HK2	hsa-miR-202	-0.9189
Anaerobic glycolysis	HK2	hsa-miR-206	-0.7051

Anaerobic glycolysis	HK2	hsa-miR-216a	-0.1677
Anaerobic glycolysis	HK2	hsa-miR-216b	-1.1704
Anaerobic glycolysis	HK2	hsa-miR-218	-0.3409
Anaerobic glycolysis	HK2	hsa-miR-28-5p	-0.1149
Anaerobic glycolysis	HK2	hsa-miR-301a	-0.2384
Anaerobic glycolysis	HK2	hsa-miR-301b	-0.2384
Anaerobic glycolysis	HK2	hsa-miR-346	-0.1208
Anaerobic glycolysis	HK2	hsa-miR-376c	-0.1505
Anaerobic glycolysis	HK2	hsa-miR-429	-0.1028
Anaerobic glycolysis	HK2	hsa-miR-495	-0.8783
Anaerobic glycolysis	HK2	hsa-miR-543	-0.8698
Anaerobic glycolysis	HK2	hsa-miR-590-3p	-0.6491
Anaerobic glycolysis	HK2	hsa-miR-599	-0.1019
Anaerobic glycolysis	HK2	hsa-miR-708	-0.1149
Anaerobic glycolysis	HK2	hsa-miR-9	-0.411
Anaerobic glycolysis	LDHA	hsa-miR-149	-0.3524
Anaerobic glycolysis	LDHA	hsa-miR-15a	-0.1626
Anaerobic glycolysis	LDHA	hsa-miR-15b	-0.1626
Anaerobic glycolysis	LDHA	hsa-miR-16	-0.1767
Anaerobic glycolysis	LDHA	hsa-miR-182	-0.9239
Anaerobic glycolysis	LDHA	hsa-miR-195	-0.1767
Anaerobic glycolysis	LDHA	hsa-miR-200b	-0.1164
Anaerobic glycolysis	LDHA	hsa-miR-200c	-0.1164
Anaerobic glycolysis	LDHA	hsa-miR-219-5p	-0.1239
Anaerobic glycolysis	LDHA	hsa-miR-296-3p	-0.185
Anaerobic glycolysis	LDHA	hsa-miR-30a	-0.1244
Anaerobic glycolysis	LDHA	hsa-miR-30b	-0.1244
Anaerobic glycolysis	LDHA	hsa-miR-30c	-0.1244
Anaerobic glycolysis	LDHA	hsa-miR-30d	-0.1256
Anaerobic glycolysis	LDHA	hsa-miR-30e	-0.1269
Anaerobic glycolysis	LDHA	hsa-miR-338-3p	-0.4434
Anaerobic glycolysis	LDHA	hsa-miR-33a	-1.1799

Anaerobic glycolysis	LDHA	hsa-miR-33b	-1.1658
Anaerobic glycolysis	LDHA	hsa-miR-34a	-0.3748
Anaerobic glycolysis	LDHA	hsa-miR-34c-5p	-0.3689
Anaerobic glycolysis	LDHA	hsa-miR-374a	-0.8928
Anaerobic glycolysis	LDHA	hsa-miR-374b	-0.886
Anaerobic glycolysis	LDHA	hsa-miR-383	-0.9034
Anaerobic glycolysis	LDHA	hsa-miR-410	-0.5588
Anaerobic glycolysis	LDHA	hsa-miR-424	-0.1626
Anaerobic glycolysis	LDHA	hsa-miR-429	-0.1176
Anaerobic glycolysis	LDHA	hsa-miR-449a	-0.3631
Anaerobic glycolysis	LDHA	hsa-miR-449b	-0.3631
Anaerobic glycolysis	LDHA	hsa-miR-497	-0.1767
Anaerobic glycolysis	LDHA	hsa-miR-503	-0.1767
Anaerobic glycolysis	LDHA	hsa-miR-590-3p	-0.8256
Anaerobic glycolysis	LDHA	hsa-miR-7	-0.1324
Anaerobic glycolysis	MCT1	hsa-miR-124	-1.255
Anaerobic glycolysis	MCT1	hsa-miR-125a-3p	-0.8453
Anaerobic glycolysis	MCT1	hsa-miR-140-5p	-0.1401
Anaerobic glycolysis	MCT1	hsa-miR-145	-0.1094
Anaerobic glycolysis	MCT1	hsa-miR-154	-0.2458
Anaerobic glycolysis	MCT1	hsa-miR-15a	-0.1429
Anaerobic glycolysis	MCT1	hsa-miR-15b	-0.1429
Anaerobic glycolysis	MCT1	hsa-miR-16	-0.1325
Anaerobic glycolysis	MCT1	hsa-miR-195	-0.1429
Anaerobic glycolysis	MCT1	hsa-miR-199a-5p	-0.116
Anaerobic glycolysis	MCT1	hsa-miR-199b-5p	-0.116
Anaerobic glycolysis	MCT1	hsa-miR-216a	-0.9523
Anaerobic glycolysis	MCT1	hsa-miR-216b	-1.0313
Anaerobic glycolysis	MCT1	hsa-miR-27a	-0.9647
Anaerobic glycolysis	MCT1	hsa-miR-27b	-0.9647
Anaerobic glycolysis	MCT1	hsa-miR-29a	-0.1138
Anaerobic glycolysis	MCT1	hsa-miR-29b	-0.1229

Anaerobic glycolysis	MCT1	hsa-miR-29c	-0.1229
Anaerobic glycolysis	MCT1	hsa-miR-320a	-0.3885
Anaerobic glycolysis	MCT1	hsa-miR-320b	-0.3885
Anaerobic glycolysis	MCT1	hsa-miR-320c	-0.3885
Anaerobic glycolysis	MCT1	hsa-miR-320d	-0.3885
Anaerobic glycolysis	MCT1	hsa-miR-324-5p	-0.764
Anaerobic glycolysis	MCT1	hsa-miR-328	-0.5792
Anaerobic glycolysis	MCT1	hsa-miR-33a	-1.1941
Anaerobic glycolysis	MCT1	hsa-miR-33b	-1.1941
Anaerobic glycolysis	MCT1	hsa-miR-342-3p	-0.7623
Anaerobic glycolysis	MCT1	hsa-miR-34a	-0.9128
Anaerobic glycolysis	MCT1	hsa-miR-34c-5p	-0.8903
Anaerobic glycolysis	MCT1	hsa-miR-371-5p	-0.6447
Anaerobic glycolysis	MCT1	hsa-miR-374a	-0.2
Anaerobic glycolysis	MCT1	hsa-miR-374b	-0.2037
Anaerobic glycolysis	MCT1	hsa-miR-377	-0.3083
Anaerobic glycolysis	MCT1	hsa-miR-384	-0.1381
Anaerobic glycolysis	MCT1	hsa-miR-424	-0.1416
Anaerobic glycolysis	MCT1	hsa-miR-425	-1.2035
Anaerobic glycolysis	MCT1	hsa-miR-449a	-0.9128
Anaerobic glycolysis	MCT1	hsa-miR-449b	-0.9128
Anaerobic glycolysis	MCT1	hsa-miR-488	-0.2368
Anaerobic glycolysis	MCT1	hsa-miR-490-3p	-0.8787
Anaerobic glycolysis	MCT1	hsa-miR-494	-0.5149
Anaerobic glycolysis	MCT1	hsa-miR-495	-0.5648
Anaerobic glycolysis	MCT1	hsa-miR-497	-0.1416
Anaerobic glycolysis	MCT1	hsa-miR-506	-1.255
Anaerobic glycolysis	MCT1	hsa-miR-539	-0.6846
Anaerobic glycolysis	MCT1	hsa-miR-590-3p	-1.1706
Anaerobic glycolysis	MCT4	hsa-miR-134	-1.1454
Anaerobic glycolysis	MCT4	hsa-miR-376a	-1.2445
Anaerobic glycolysis	MCT4	hsa-miR-376b	-1.2445

Anaerobic glycolysis	MCT4	hsa-miR-377	-0.1594
Anaerobic glycolysis	MCT4	hsa-miR-485-5p	-0.136
Anaerobic glycolysis	MCT4	hsa-miR-590-3p	-0.6284
Anaerobic glycolysis	MCT4	hsa-miR-876-5p	-0.1302
Anaerobic glycolysis	PGAM1	hsa-miR-135a	-0.9636
Anaerobic glycolysis	PGAM1	hsa-miR-135b	-0.9636
Anaerobic glycolysis	PGAM1	hsa-miR-141	-0.1077
Anaerobic glycolysis	PGAM1	hsa-miR-190	-0.3519
Anaerobic glycolysis	PGAM1	hsa-miR-190b	-0.3491
Anaerobic glycolysis	PGAM1	hsa-miR-197	-0.1519
Anaerobic glycolysis	PGAM1	hsa-miR-200a	-0.1077
Anaerobic glycolysis	PGAM1	hsa-miR-224	-0.346
Anaerobic glycolysis	PGAM1	hsa-miR-25	-1.0933
Anaerobic glycolysis	PGAM1	hsa-miR-28-5p	-0.3563
Anaerobic glycolysis	PGAM1	hsa-miR-299-3p	-0.7931
Anaerobic glycolysis	PGAM1	hsa-miR-30a	-0.5051
Anaerobic glycolysis	PGAM1	hsa-miR-30b	-0.4982
Anaerobic glycolysis	PGAM1	hsa-miR-30c	-0.4982
Anaerobic glycolysis	PGAM1	hsa-miR-30d	-0.5051
Anaerobic glycolysis	PGAM1	hsa-miR-30e	-0.5051
Anaerobic glycolysis	PGAM1	hsa-miR-32	-1.0957
Anaerobic glycolysis	PGAM1	hsa-miR-326	-0.1637
Anaerobic glycolysis	PGAM1	hsa-miR-329	-0.4883
Anaerobic glycolysis	PGAM1	hsa-miR-330-5p	-0.1653
Anaerobic glycolysis	PGAM1	hsa-miR-34a	-0.1093
Anaerobic glycolysis	PGAM1	hsa-miR-34c-5p	-0.1022
Anaerobic glycolysis	PGAM1	hsa-miR-362-3p	-0.4589
Anaerobic glycolysis	PGAM1	hsa-miR-363	-1.0909
Anaerobic glycolysis	PGAM1	hsa-miR-367	-1.1004
Anaerobic glycolysis	PGAM1	hsa-miR-449a	-0.1115
Anaerobic glycolysis	PGAM1	hsa-miR-449b	-0.1115
Anaerobic glycolysis	PGAM1	hsa-miR-708	-0.3563

Anaerobic glycolysis	PGAM1	hsa-miR-876-5p	-0.1901
Anaerobic glycolysis	PGAM1	hsa-miR-92a	-1.0957
Anaerobic glycolysis	PGAM1	hsa-miR-92b	-1.0957
Anaerobic glycolysis	PKM2	hsa-miR-122	-0.6311
Anaerobic glycolysis	PKM2	hsa-miR-148a	-0.3262
Anaerobic glycolysis	PKM2	hsa-miR-149	-0.1549
Anaerobic glycolysis	PKM2	hsa-miR-19a	-0.1251
Anaerobic glycolysis	PKM2	hsa-miR-19b	-0.1251
Anaerobic glycolysis	PKM2	hsa-miR-326	-0.2015
Anaerobic glycolysis	PKM2	hsa-miR-330-5p	-0.2072
Anaerobic glycolysis	PKM2	hsa-miR-338-3p	-0.4546
Anaerobic glycolysis	PKM2	hsa-miR-491-5p	-0.1114
de novo fatty acid synthesis	ACC1	hsa-miR-1	-0.1794
de novo fatty acid synthesis	ACC1	hsa-miR-134	-0.2403
de novo fatty acid synthesis	ACC1	hsa-miR-139-5p	-0.1611
de novo fatty acid synthesis	ACC1	hsa-miR-140-5p	-0.4045
de novo fatty acid synthesis	ACC1	hsa-miR-142-3p	-0.111
de novo fatty acid synthesis	ACC1	hsa-miR-149	-0.17
de novo fatty acid synthesis	ACC1	hsa-miR-190	-0.1852
de novo fatty acid synthesis	ACC1	hsa-miR-190b	-0.1834
de novo fatty acid synthesis	ACC1	hsa-miR-19a	-0.3135
de novo fatty acid synthesis	ACC1	hsa-miR-19b	-0.3343
de novo fatty acid synthesis	ACC1	hsa-miR-200b	-0.5089
de novo fatty acid synthesis	ACC1	hsa-miR-200c	-0.5089
de novo fatty acid synthesis	ACC1	hsa-miR-206	-0.1777
de novo fatty acid synthesis	ACC1	hsa-miR-24	-0.4487
de novo fatty acid synthesis	ACC1	hsa-miR-27a	-0.2334
de novo fatty acid synthesis	ACC1	hsa-miR-27b	-0.2334
de novo fatty acid synthesis	ACC1	hsa-miR-28-5p	-0.107
de novo fatty acid synthesis	ACC1	hsa-miR-299-3p	-0.9764
de novo fatty acid synthesis	ACC1	hsa-miR-29a	-0.2044
de novo fatty acid synthesis	ACC1	hsa-miR-29b	-0.2044

de novo fatty acid synthesis	ACC1	hsa-miR-29c	-0.2044
de novo fatty acid synthesis	ACC1	hsa-miR-378	-0.118
de novo fatty acid synthesis	ACC1	hsa-miR-421	-0.1058
de novo fatty acid synthesis	ACC1	hsa-miR-422a	-0.118
de novo fatty acid synthesis	ACC1	hsa-miR-429	-0.5089
de novo fatty acid synthesis	ACC1	hsa-miR-491-5p	-0.2842
de novo fatty acid synthesis	ACC1	hsa-miR-494	-0.2471
de novo fatty acid synthesis	ACC1	hsa-miR-495	-0.2093
de novo fatty acid synthesis	ACC1	hsa-miR-599	-0.7898
de novo fatty acid synthesis	ACC1	hsa-miR-613	-0.1777
de novo fatty acid synthesis	ACC1	hsa-miR-653	-0.4641
de novo fatty acid synthesis	ACC1	hsa-miR-708	-0.1091
de novo fatty acid synthesis	ACC1	hsa-miR-9	-0.1236
de novo fatty acid synthesis	ACLY	hsa-miR-101	-0.6006
de novo fatty acid synthesis	ACLY	hsa-miR-149	-0.1931
de novo fatty acid synthesis	ACLY	hsa-miR-214	-1.0517
de novo fatty acid synthesis	ACLY	hsa-miR-22	-0.161
de novo fatty acid synthesis	ACLY	hsa-miR-224	-1.0445
de novo fatty acid synthesis	ACLY	hsa-miR-27a	-0.1623
de novo fatty acid synthesis	ACLY	hsa-miR-27b	-0.1623
de novo fatty acid synthesis	ACLY	hsa-miR-340	-0.1743
de novo fatty acid synthesis	ACLY	hsa-miR-374a	-0.8706
de novo fatty acid synthesis	ACLY	hsa-miR-374b	-0.8637
de novo fatty acid synthesis	ACLY	hsa-miR-544	-1.1463
de novo fatty acid synthesis	ACLY	hsa-miR-758	-1.2361
de novo fatty acid synthesis	CIC	hsa-miR-1271	-0.6959
de novo fatty acid synthesis	CIC	hsa-miR-185	-0.105
de novo fatty acid synthesis	CIC	hsa-miR-96	-0.4515
de novo fatty acid synthesis	FASN	hsa-miR-103	-0.5702
de novo fatty acid synthesis	FASN	hsa-miR-107	-0.5702
de novo fatty acid synthesis	FASN	hsa-miR-15a	-0.9849
de novo fatty acid synthesis	FASN	hsa-miR-15b	-0.9643

de novo fatty acid synthesis	FASN	hsa-miR-16	-0.9819
de novo fatty acid synthesis	FASN	hsa-miR-195	-0.9819
de novo fatty acid synthesis	FASN	hsa-miR-424	-0.9879
de novo fatty acid synthesis	FASN	hsa-miR-485-5p	-0.3113
de novo fatty acid synthesis	FASN	hsa-miR-495	-0.9163
de novo fatty acid synthesis	FASN	hsa-miR-497	-0.9849
de novo fatty acid synthesis	SCD	hsa-let-7a	-0.3791
de novo fatty acid synthesis	SCD	hsa-let-7b	-0.3821
de novo fatty acid synthesis	SCD	hsa-let-7c	-0.3791
de novo fatty acid synthesis	SCD	hsa-let-7d	-0.3851
de novo fatty acid synthesis	SCD	hsa-let-7e	-0.3791
de novo fatty acid synthesis	SCD	hsa-let-7f	-0.3821
de novo fatty acid synthesis	SCD	hsa-let-7g	-0.3791
de novo fatty acid synthesis	SCD	hsa-let-7i	-0.3821
de novo fatty acid synthesis	SCD	hsa-miR-124	-0.1232
de novo fatty acid synthesis	SCD	hsa-miR-141	-0.1278
de novo fatty acid synthesis	SCD	hsa-miR-142-3p	-0.1429
de novo fatty acid synthesis	SCD	hsa-miR-150	-0.3043
de novo fatty acid synthesis	SCD	hsa-miR-181a	-1.0566
de novo fatty acid synthesis	SCD	hsa-miR-181b	-1.0566
de novo fatty acid synthesis	SCD	hsa-miR-181c	-1.0592
de novo fatty acid synthesis	SCD	hsa-miR-181d	-1.0566
de novo fatty acid synthesis	SCD	hsa-miR-185	-0.1263
de novo fatty acid synthesis	SCD	hsa-miR-186	-0.6811
de novo fatty acid synthesis	SCD	hsa-miR-192	-0.419
de novo fatty acid synthesis	SCD	hsa-miR-200a	-0.1278
de novo fatty acid synthesis	SCD	hsa-miR-200b	-0.6415
de novo fatty acid synthesis	SCD	hsa-miR-200c	-0.6415
de novo fatty acid synthesis	SCD	hsa-miR-204	-0.3049
de novo fatty acid synthesis	SCD	hsa-miR-211	-0.3049
de novo fatty acid synthesis	SCD	hsa-miR-214	-0.1159
de novo fatty acid synthesis	SCD	hsa-miR-215	-0.419

de novo fatty acid synthesis	SCD	hsa-miR-216a	-0.4374
de novo fatty acid synthesis	SCD	hsa-miR-22	-0.1226
de novo fatty acid synthesis	SCD	hsa-miR-324-5p	-0.2347
de novo fatty acid synthesis	SCD	hsa-miR-346	-0.1795
de novo fatty acid synthesis	SCD	hsa-miR-382	-0.6255
de novo fatty acid synthesis	SCD	hsa-miR-383	-0.3388
de novo fatty acid synthesis	SCD	hsa-miR-429	-0.6452
de novo fatty acid synthesis	SCD	hsa-miR-433	-0.4409
de novo fatty acid synthesis	SCD	hsa-miR-495	-0.763
de novo fatty acid synthesis	SCD	hsa-miR-499-5p	-0.2084
de novo fatty acid synthesis	SCD	hsa-miR-504	-0.136
de novo fatty acid synthesis	SCD	hsa-miR-506	-0.1207
de novo fatty acid synthesis	SCD	hsa-miR-539	-0.4766
de novo fatty acid synthesis	SCD	hsa-miR-590-3p	-0.4403
de novo fatty acid synthesis	SCD	hsa-miR-599	-0.5856
de novo fatty acid synthesis	SCD	hsa-miR-98	-0.3791
Gluconeogenesis	PCK1	hsa-miR-101	-1.247
Gluconeogenesis	PCK1	hsa-miR-129-5p	-0.1417
Gluconeogenesis	PCK1	hsa-miR-1297	-0.9024
Gluconeogenesis	PCK1	hsa-miR-132	-0.2238
Gluconeogenesis	PCK1	hsa-miR-137	-0.1063
Gluconeogenesis	PCK1	hsa-miR-141	-0.9202
Gluconeogenesis	PCK1	hsa-miR-144	-0.241
Gluconeogenesis	PCK1	hsa-miR-199a-5p	-0.5688
Gluconeogenesis	PCK1	hsa-miR-199b-5p	-0.5724
Gluconeogenesis	PCK1	hsa-miR-200a	-0.9235
Gluconeogenesis	PCK1	hsa-miR-204	-0.1178
Gluconeogenesis	PCK1	hsa-miR-211	-0.1079
Gluconeogenesis	PCK1	hsa-miR-212	-0.2238
Gluconeogenesis	PCK1	hsa-miR-25	-0.2659
Gluconeogenesis	PCK1	hsa-miR-26a	-0.9024
Gluconeogenesis	PCK1	hsa-miR-26b	-0.9024

Gluconeogenesis	PCK1	hsa-miR-32	-0.2613
Gluconeogenesis	PCK1	hsa-miR-33a	-0.7617
Gluconeogenesis	PCK1	hsa-miR-33b	-0.7617
Gluconeogenesis	PCK1	hsa-miR-340	-0.1579
Gluconeogenesis	PCK1	hsa-miR-363	-0.2636
Gluconeogenesis	PCK1	hsa-miR-367	-0.2613
Gluconeogenesis	PCK1	hsa-miR-495	-0.9769
Gluconeogenesis	PCK1	hsa-miR-590-3p	-0.1438
Gluconeogenesis	PCK1	hsa-miR-92a	-0.2659
Gluconeogenesis	PCK1	hsa-miR-92b	-0.2659
Gluconeogenesis	PCK2	hsa-miR-183	-1.195
Gluconeogenesis	PCK2	hsa-miR-18a	-0.2951
Gluconeogenesis	PCK2	hsa-miR-18b	-0.2951
Gluconeogenesis	PCK2	hsa-miR-544	-1.0475
Glutaminolysis	GLS1	hsa-miR-103	-0.143
Glutaminolysis	GLS1	hsa-miR-107	-0.143
Glutaminolysis	GLS1	hsa-miR-10a	-0.8715
Glutaminolysis	GLS1	hsa-miR-10b	-0.8715
Glutaminolysis	GLS1	hsa-miR-125a-5p	-0.107
Glutaminolysis	GLS1	hsa-miR-125b	-0.107
Glutaminolysis	GLS1	hsa-miR-137	-0.6129
Glutaminolysis	GLS1	hsa-miR-141	-0.9338
Glutaminolysis	GLS1	hsa-miR-153	-0.7207
Glutaminolysis	GLS1	hsa-miR-15a	-0.141
Glutaminolysis	GLS1	hsa-miR-15b	-0.141
Glutaminolysis	GLS1	hsa-miR-16	-0.141
Glutaminolysis	GLS1	hsa-miR-181a	-0.7141
Glutaminolysis	GLS1	hsa-miR-181b	-0.7141
Glutaminolysis	GLS1	hsa-miR-181c	-0.7141
Glutaminolysis	GLS1	hsa-miR-181d	-0.7141
Glutaminolysis	GLS1	hsa-miR-186	-0.3993
Glutaminolysis	GLS1	hsa-miR-187	-0.6671

Glutaminolysis	GLS1	hsa-miR-18a	-0.3239
Glutaminolysis	GLS1	hsa-miR-18b	-0.3239
Glutaminolysis	GLS1	hsa-miR-195	-0.141
Glutaminolysis	GLS1	hsa-miR-200a	-0.937
Glutaminolysis	GLS1	hsa-miR-202	-0.1324
Glutaminolysis	GLS1	hsa-miR-203	-0.5873
Glutaminolysis	GLS1	hsa-miR-216b	-0.2413
Glutaminolysis	GLS1	hsa-miR-217	-0.7204
Glutaminolysis	GLS1	hsa-miR-218	-0.472
Glutaminolysis	GLS1	hsa-miR-224	-0.5338
Glutaminolysis	GLS1	hsa-miR-23a	-0.7464
Glutaminolysis	GLS1	hsa-miR-23b	-0.7464
Glutaminolysis	GLS1	hsa-miR-301a	-0.493
Glutaminolysis	GLS1	hsa-miR-340	-0.4758
Glutaminolysis	GLS1	hsa-miR-342-3p	-0.1025
Glutaminolysis	GLS1	hsa-miR-361-5p	-0.3301
Glutaminolysis	GLS1	hsa-miR-376a	-0.4936
Glutaminolysis	GLS1	hsa-miR-376b	-0.4936
Glutaminolysis	GLS1	hsa-miR-377	-0.9708
Glutaminolysis	GLS1	hsa-miR-378	-0.4741
Glutaminolysis	GLS1	hsa-miR-383	-0.8261
Glutaminolysis	GLS1	hsa-miR-422a	-0.4708
Glutaminolysis	GLS1	hsa-miR-424	-0.1396
Glutaminolysis	GLS1	hsa-miR-448	-0.4431
Glutaminolysis	GLS1	hsa-miR-454	-0.4999
Glutaminolysis	GLS1	hsa-miR-485-5p	-0.4225
Glutaminolysis	GLS1	hsa-miR-495	-0.1374
Glutaminolysis	GLS1	hsa-miR-497	-0.1369
Glutaminolysis	GLS1	hsa-miR-543	-0.92
Glutaminolysis	GLS1	hsa-miR-7	-0.6608
Glutaminolysis	GLS1	hsa-miR-9	-0.7217
Glutaminolysis	GLS2	hsa-miR-103	-0.2256

Glutaminolysis	GLS2	hsa-miR-107	-0.2256
Glutaminolysis	GLS2	hsa-miR-133a	-0.1283
Glutaminolysis	GLS2	hsa-miR-133b	-0.1283
Glutaminolysis	GLS2	hsa-miR-15a	-0.2986
Glutaminolysis	GLS2	hsa-miR-15b	-0.296
Glutaminolysis	GLS2	hsa-miR-16	-0.7886
Glutaminolysis	GLS2	hsa-miR-190	-0.6178
Glutaminolysis	GLS2	hsa-miR-190b	-0.6215
Glutaminolysis	GLS2	hsa-miR-195	-0.8165
Glutaminolysis	GLS2	hsa-miR-200b	-0.1085
Glutaminolysis	GLS2	hsa-miR-200c	-0.1085
Glutaminolysis	GLS2	hsa-miR-224	-0.4343
Glutaminolysis	GLS2	hsa-miR-424	-0.7949
Glutaminolysis	GLS2	hsa-miR-429	-0.1074
Glutaminolysis	GLS2	hsa-miR-433	-0.178
Glutaminolysis	GLS2	hsa-miR-497	-0.8058
PPP pathway	G6PD	hsa-miR-1	-0.1707
PPP pathway	G6PD	hsa-miR-138	-0.8302
PPP pathway	G6PD	hsa-miR-206	-0.169
PPP pathway	G6PD	hsa-miR-326	-0.2014
PPP pathway	G6PD	hsa-miR-330-5p	-0.1977
PPP pathway	G6PD	hsa-miR-485-5p	-0.1766
PPP pathway	G6PD	hsa-miR-613	-0.1807
PPP pathway	TKTL1	hsa-miR-101	-0.7228
PPP pathway	TKTL1	hsa-miR-125a-3p	-0.2799
PPP pathway	TKTL1	hsa-miR-143	-0.3409
PPP pathway	TKTL1	hsa-miR-15a	-0.2121
PPP pathway	TKTL1	hsa-miR-15b	-0.2102
PPP pathway	TKTL1	hsa-miR-16	-0.2102
PPP pathway	TKTL1	hsa-miR-195	-0.2102
PPP pathway	TKTL1	hsa-miR-203	-1.1436
PPP pathway	TKTL1	hsa-miR-204	-0.8089

PPP pathway	TKTL1	hsa-miR-211	-0.8125
PPP pathway	TKTL1	hsa-miR-361-5p	-1.1944
PPP pathway	TKTL1	hsa-miR-371-5p	-0.376
PPP pathway	TKTL1	hsa-miR-382	-0.6705
PPP pathway	TKTL1	hsa-miR-424	-0.2102
PPP pathway	TKTL1	hsa-miR-448	-0.4531
PPP pathway	TKTL1	hsa-miR-495	-0.6947
PPP pathway	TKTL1	hsa-miR-497	-0.2141
PPP pathway	TKTL1	hsa-miR-542-3p	-1.2302
PPP pathway	TKTL1	hsa-miR-543	-0.2017
Serine, glycine and one carbon metabolism	GNMT	hsa-miR-873	-0.842
Serine, glycine and one carbon metabolism	MTHFD1L	hsa-miR-106a	-0.2715
Serine, glycine and one carbon metabolism	MTHFD1L	hsa-miR-106b	-0.2715
Serine, glycine and one carbon metabolism	MTHFD1L	hsa-miR-17	-0.2715
Serine, glycine and one carbon metabolism	MTHFD1L	hsa-miR-181a	-0.2774
Serine, glycine and one carbon metabolism	MTHFD1L	hsa-miR-181b	-0.2774
Serine, glycine and one carbon metabolism	MTHFD1L	hsa-miR-181c	-1.2521
Serine, glycine and one carbon metabolism	MTHFD1L	hsa-miR-181d	-0.2774
Serine, glycine and one carbon metabolism	MTHFD1L	hsa-miR-20a	-0.2739
Serine, glycine and one carbon metabolism	MTHFD1L	hsa-miR-20b	-0.2739
Serine, glycine and one carbon metabolism	MTHFD1L	hsa-miR-338-3p	-1.1865
Serine, glycine and one carbon metabolism	MTHFD1L	hsa-miR-377	-0.101
Serine, glycine and one carbon metabolism	MTHFD1L	hsa-miR-411	-0.1837
Serine, glycine and one carbon metabolism	MTHFD1L	hsa-miR-448	-0.1014
Serine, glycine and one carbon metabolism	MTHFD1L	hsa-miR-494	-0.1277
Serine, glycine and one carbon metabolism	MTHFD1L	hsa-miR-519d	-0.2762
Serine, glycine and one carbon metabolism	MTHFD1L	hsa-miR-543	-1.2796
Serine, glycine and one carbon metabolism	MTHFD1L	hsa-miR-9	-0.1902
Serine, glycine and one carbon metabolism	MTHFD1L	hsa-miR-93	-0.2787
Serine, glycine and one carbon metabolism	MTHFD2	hsa-miR-124	-0.1515
Serine, glycine and one carbon metabolism	MTHFD2	hsa-miR-137	-0.2296
Serine, glycine and one carbon metabolism	MTHFD2	hsa-miR-146a	-0.8223

Serine, glycine and one carbon metabolism	MTHFD2	hsa-miR-146b-5p	-0.8223
Serine, glycine and one carbon metabolism	MTHFD2	hsa-miR-186	-0.4257
Serine, glycine and one carbon metabolism	MTHFD2	hsa-miR-202	-0.1775
Serine, glycine and one carbon metabolism	MTHFD2	hsa-miR-22	-0.4548
Serine, glycine and one carbon metabolism	MTHFD2	hsa-miR-25	-0.2307
Serine, glycine and one carbon metabolism	MTHFD2	hsa-miR-300	-0.8644
Serine, glycine and one carbon metabolism	MTHFD2	hsa-miR-32	-0.2307
Serine, glycine and one carbon metabolism	MTHFD2	hsa-miR-33a	-0.1969
Serine, glycine and one carbon metabolism	MTHFD2	hsa-miR-33b	-0.1969
Serine, glycine and one carbon metabolism	MTHFD2	hsa-miR-340	-0.1386
Serine, glycine and one carbon metabolism	MTHFD2	hsa-miR-363	-0.2371
Serine, glycine and one carbon metabolism	MTHFD2	hsa-miR-367	-0.2371
Serine, glycine and one carbon metabolism	MTHFD2	hsa-miR-371-5p	-1.2424
Serine, glycine and one carbon metabolism	MTHFD2	hsa-miR-381	-0.8644
Serine, glycine and one carbon metabolism	MTHFD2	hsa-miR-383	-0.188
Serine, glycine and one carbon metabolism	MTHFD2	hsa-miR-384	-0.7498
Serine, glycine and one carbon metabolism	MTHFD2	hsa-miR-410	-0.2204
Serine, glycine and one carbon metabolism	MTHFD2	hsa-miR-411	-0.2435
Serine, glycine and one carbon metabolism	MTHFD2	hsa-miR-421	-0.8609
Serine, glycine and one carbon metabolism	MTHFD2	hsa-miR-496	-0.3163
Serine, glycine and one carbon metabolism	MTHFD2	hsa-miR-504	-0.2212
Serine, glycine and one carbon metabolism	MTHFD2	hsa-miR-505	-0.8269
Serine, glycine and one carbon metabolism	MTHFD2	hsa-miR-506	-0.1529
Serine, glycine and one carbon metabolism	MTHFD2	hsa-miR-544	0.2589
Serine, glycine and one carbon metabolism	MTHFD2	hsa-miR-590-3p	-0.2199
Serine, glycine and one carbon metabolism	MTHFD2	hsa-miR-758	-0.6521
Serine, glycine and one carbon metabolism	MTHFD2	hsa-miR-9	-0.9973
Serine, glycine and one carbon metabolism	MTHFD2	hsa-miR-92a	-0.2349
Serine, glycine and one carbon metabolism	MTHFD2	hsa-miR-92b	-0.2349
Serine, glycine and one carbon metabolism	PHGDH	hsa-miR-128	-0.4307
Serine, glycine and one carbon metabolism	PHGDH	hsa-miR-137	-0.6362
Serine, glycine and one carbon metabolism	PHGDH	hsa-miR-27a	-0.2951

Serine, glycine and one carbon metabolism	PHGDH	hsa-miR-27b	-0.2951
Serine, glycine and one carbon metabolism	PSAT1	hsa-miR-139-5p	-0.7778
Serine, glycine and one carbon metabolism	PSAT1	hsa-miR-145	-0.7431
Serine, glycine and one carbon metabolism	PSAT1	hsa-miR-15a	-1.1188
Serine, glycine and one carbon metabolism	PSAT1	hsa-miR-15b	-1.1166
Serine, glycine and one carbon metabolism	PSAT1	hsa-miR-16	-1.1098
Serine, glycine and one carbon metabolism	PSAT1	hsa-miR-183	-0.1102
Serine, glycine and one carbon metabolism	PSAT1	hsa-miR-186	-0.2118
Serine, glycine and one carbon metabolism	PSAT1	hsa-miR-195	-1.1098
Serine, glycine and one carbon metabolism	PSAT1	hsa-miR-200b	-1.1237
Serine, glycine and one carbon metabolism	PSAT1	hsa-miR-200c	-1.1237
Serine, glycine and one carbon metabolism	PSAT1	hsa-miR-203	-0.8792
Serine, glycine and one carbon metabolism	PSAT1	hsa-miR-20b	-0.9028
Serine, glycine and one carbon metabolism	PSAT1	hsa-miR-340	-1.2017
Serine, glycine and one carbon metabolism	PSAT1	hsa-miR-410	-0.1258
Serine, glycine and one carbon metabolism	PSAT1	hsa-miR-411	-0.1001
Serine, glycine and one carbon metabolism	PSAT1	hsa-miR-421	-0.2786
Serine, glycine and one carbon metabolism	PSAT1	hsa-miR-424	-1.1166
Serine, glycine and one carbon metabolism	PSAT1	hsa-miR-429	-1.1215
Serine, glycine and one carbon metabolism	PSAT1	hsa-miR-497	-1.121
Serine, glycine and one carbon metabolism	PSPH	hsa-miR-139-5p	-1.2067
Serine, glycine and one carbon metabolism	PSPH	hsa-miR-144	-0.649
Serine, glycine and one carbon metabolism	PSPH	hsa-miR-186	-1.0138
Serine, glycine and one carbon metabolism	PSPH	hsa-miR-200b	-1.0781
Serine, glycine and one carbon metabolism	PSPH	hsa-miR-200c	-1.0781
Serine, glycine and one carbon metabolism	PSPH	hsa-miR-221	-0.1714
Serine, glycine and one carbon metabolism	PSPH	hsa-miR-222	-0.1682
Serine, glycine and one carbon metabolism	PSPH	hsa-miR-374a	-0.6258
Serine, glycine and one carbon metabolism	PSPH	hsa-miR-374b	-0.6332
Serine, glycine and one carbon metabolism	PSPH	hsa-miR-382	-1.0781
Serine, glycine and one carbon metabolism	PSPH	hsa-miR-429	-1.0756
Serine, glycine and one carbon metabolism	PSPH	hsa-miR-485-5p	-0.1808

Serine, glycine and one carbon metabolism	SHMT1	hsa-miR-129-5p	-0.5572
Serine, glycine and one carbon metabolism	SHMT1	hsa-miR-181a	-0.151
Serine, glycine and one carbon metabolism	SHMT1	hsa-miR-181b	-0.151
Serine, glycine and one carbon metabolism	SHMT1	hsa-miR-181c	-0.151
Serine, glycine and one carbon metabolism	SHMT1	hsa-miR-181d	-0.1496
Serine, glycine and one carbon metabolism	SHMT1	hsa-miR-18a	-0.5701
Serine, glycine and one carbon metabolism	SHMT1	hsa-miR-203	-0.8138
Serine, glycine and one carbon metabolism	SHMT1	hsa-miR-218	-1.3314
Serine, glycine and one carbon metabolism	SHMT1	hsa-miR-219-5p	-0.4498
Serine, glycine and one carbon metabolism	SHMT1	hsa-miR-222	-0.5936
Serine, glycine and one carbon metabolism	SHMT1	hsa-miR-320a	-0.6858
Serine, glycine and one carbon metabolism	SHMT1	hsa-miR-320b	-0.6858
Serine, glycine and one carbon metabolism	SHMT1	hsa-miR-320c	-0.6858
Serine, glycine and one carbon metabolism	SHMT1	hsa-miR-320d	-0.6858
Serine, glycine and one carbon metabolism	SHMT1	hsa-miR-340	-0.3981
Serine, glycine and one carbon metabolism	SHMT1	hsa-miR-34a	-0.1715
Serine, glycine and one carbon metabolism	SHMT1	hsa-miR-34c-5p	-0.1682
Serine, glycine and one carbon metabolism	SHMT1	hsa-miR-374a	-0.1518
Serine, glycine and one carbon metabolism	SHMT1	hsa-miR-374b	-0.1518
Serine, glycine and one carbon metabolism	SHMT1	hsa-miR-384	-1.2356
Serine, glycine and one carbon metabolism	SHMT1	hsa-miR-410	-0.3556
Serine, glycine and one carbon metabolism	SHMT1	hsa-miR-421	-0.1834
Serine, glycine and one carbon metabolism	SHMT1	hsa-miR-449a	-0.1715
Serine, glycine and one carbon metabolism	SHMT1	hsa-miR-449b	-0.1715
Serine, glycine and one carbon metabolism	SHMT1	hsa-miR-505	-0.1719
Serine, glycine and one carbon metabolism	SHMT1	hsa-miR-9	-0.1026
Serine, glycine and one carbon metabolism	SHMT2	hsa-miR-149	-0.6624
Serine, glycine and one carbon metabolism	SHMT2	hsa-miR-193a-3p	-0.4466
Serine, glycine and one carbon metabolism	SHMT2	hsa-miR-193b	-0.4466
Serine, glycine and one carbon metabolism	SHMT2	hsa-miR-300	-0.151
Serine, glycine and one carbon metabolism	SHMT2	hsa-miR-381	-0.151
Serine, glycine and one carbon metabolism	SHMT2	hsa-miR-383	-0.8414

Serine, glycine and one carbon metabolism	SHMT2	hsa-miR-485-5p	-0.2113
Serine, glycine and one carbon metabolism	SHMT2	hsa-miR-495	-0.2878
TCA cycle	FH	hsa-miR-296-3p	-0.1178
TCA cycle	FH	hsa-miR-340	-0.3979
TCA cycle	FH	hsa-miR-590-3p	-1.2189
TCA cycle	IDH1	hsa-miR-106a	-0.8261
TCA cycle	IDH1	hsa-miR-106b	-0.8261
TCA cycle	IDH1	hsa-miR-132	-0.2548
TCA cycle	IDH1	hsa-miR-133a	-0.3898
TCA cycle	IDH1	hsa-miR-133b	-0.3898
TCA cycle	IDH1	hsa-miR-137	-0.1519
TCA cycle	IDH1	hsa-miR-144	-0.7462
TCA cycle	IDH1	hsa-miR-149	-0.2058
TCA cycle	IDH1	hsa-miR-17	-0.8261
TCA cycle	IDH1	hsa-miR-181a	-0.4734
TCA cycle	IDH1	hsa-miR-181b	-0.4734
TCA cycle	IDH1	hsa-miR-181c	-0.4734
TCA cycle	IDH1	hsa-miR-181d	-0.4734
TCA cycle	IDH1	hsa-miR-186	-0.3296
TCA cycle	IDH1	hsa-miR-18a	-0.807
TCA cycle	IDH1	hsa-miR-194	-0.1182
TCA cycle	IDH1	hsa-miR-196a	-0.6086
TCA cycle	IDH1	hsa-miR-196b	-0.6086
TCA cycle	IDH1	hsa-miR-200b	-0.5748
TCA cycle	IDH1	hsa-miR-200c	-0.6031
TCA cycle	IDH1	hsa-miR-204	-0.1026
TCA cycle	IDH1	hsa-miR-21	-0.171
TCA cycle	IDH1	hsa-miR-212	-0.2548
TCA cycle	IDH1	hsa-miR-23a	-1.2987
TCA cycle	IDH1	hsa-miR-23b	-1.2987
TCA cycle	IDH1	hsa-miR-25	-1.2827
TCA cycle	IDH1	hsa-miR-30a	-0.2337

TCA cycle	IDH1	hsa-miR-30b	-0.2337
TCA cycle	IDH1	hsa-miR-30c	-0.2337
TCA cycle	IDH1	hsa-miR-30d	-0.2358
TCA cycle	IDH1	hsa-miR-30e	-0.2337
TCA cycle	IDH1	hsa-miR-32	-1.2809
TCA cycle	IDH1	hsa-miR-326	-0.2983
TCA cycle	IDH1	hsa-miR-330-5p	-0.3009
TCA cycle	IDH1	hsa-miR-338-3p	-0.1226
TCA cycle	IDH1	hsa-miR-340	-0.2232
TCA cycle	IDH1	hsa-miR-34a	-0.7258
TCA cycle	IDH1	hsa-miR-34c-5p	-0.7258
TCA cycle	IDH1	hsa-miR-363	-1.2809
TCA cycle	IDH1	hsa-miR-367	-1.2791
TCA cycle	IDH1	hsa-miR-374a	-1.1396
TCA cycle	IDH1	hsa-miR-374b	-1.1437
TCA cycle	IDH1	hsa-miR-384	-0.3022
TCA cycle	IDH1	hsa-miR-410	-0.4165
TCA cycle	IDH1	hsa-miR-421	-1.2709
TCA cycle	IDH1	hsa-miR-425	-0.5891
TCA cycle	IDH1	hsa-miR-429	-0.3898
TCA cycle	IDH1	hsa-miR-448	-1.0867
TCA cycle	IDH1	hsa-miR-449a	-0.7258
TCA cycle	IDH1	hsa-miR-449b	-0.7258
TCA cycle	IDH1	hsa-miR-494	-0.7487
TCA cycle	IDH1	hsa-miR-539	-1.2299
TCA cycle	IDH1	hsa-miR-542-3p	-1.0827
TCA cycle	IDH1	hsa-miR-543	-0.6643
TCA cycle	IDH1	hsa-miR-590-3p	-1.2775
TCA cycle	IDH1	hsa-miR-590-5p	-0.1603
TCA cycle	IDH1	hsa-miR-92a	-1.2836
TCA cycle	IDH1	hsa-miR-92b	-1.2836
TCA cycle	IDH1	hsa-miR-93	-0.8367

TCA cycle	IDH2	hsa-miR-144	-0.6488
TCA cycle	IDH2	hsa-miR-149	-0.3653
TCA cycle	IDH2	hsa-miR-183	-1.1327
TCA cycle	ME1	hsa-miR-129-5p	-0.1796
TCA cycle	ME1	hsa-miR-133a	-0.1982
TCA cycle	ME1	hsa-miR-133b	-0.1982
TCA cycle	ME1	hsa-miR-142-3p	-0.9845
TCA cycle	ME1	hsa-miR-143	-0.1386
TCA cycle	ME1	hsa-miR-144	-0.1178
TCA cycle	ME1	hsa-miR-146a	-0.1028
TCA cycle	ME1	hsa-miR-146b-5p	-0.1028
TCA cycle	ME1	hsa-miR-153	-1.0713
TCA cycle	ME1	hsa-miR-186	-0.9513
TCA cycle	ME1	hsa-miR-223	-0.1249
TCA cycle	ME1	hsa-miR-25	-0.9932
TCA cycle	ME1	hsa-miR-30a	-0.652
TCA cycle	ME1	hsa-miR-30b	-0.6234
TCA cycle	ME1	hsa-miR-30c	-0.6234
TCA cycle	ME1	hsa-miR-30d	-0.6558
TCA cycle	ME1	hsa-miR-30e	-0.6483
TCA cycle	ME1	hsa-miR-32	-0.9932
TCA cycle	ME1	hsa-miR-363	-0.9902
TCA cycle	ME1	hsa-miR-367	-0.9872
TCA cycle	ME1	hsa-miR-374a	-0.5051
TCA cycle	ME1	hsa-miR-374b	-0.4982
TCA cycle	ME1	hsa-miR-376a	-0.6838
TCA cycle	ME1	hsa-miR-376b	-0.6838
TCA cycle	ME1	hsa-miR-376c	-0.8172
TCA cycle	ME1	hsa-miR-410	-0.3724
TCA cycle	ME1	hsa-miR-411	-0.1092
TCA cycle	ME1	hsa-miR-421	-0.1016
TCA cycle	ME1	hsa-miR-448	-0.142

TCA cycle	ME1	hsa-miR-486-5p	-0.1323
TCA cycle	ME1	hsa-miR-488	-1.25
TCA cycle	ME1	hsa-miR-505	-1.1298
TCA cycle	ME1	hsa-miR-599	-0.1409
TCA cycle	ME1	hsa-miR-876-5p	-0.4419
TCA cycle	ME1	hsa-miR-92a	-0.9961
TCA cycle	ME1	hsa-miR-92b	-0.9961
TCA cycle	PDHX	hsa-miR-1271	-0.1695
TCA cycle	PDHX	hsa-miR-128	-1.2851
TCA cycle	PDHX	hsa-miR-129-5p	-0.1265
TCA cycle	PDHX	hsa-miR-1297	-1.2696
TCA cycle	PDHX	hsa-miR-135a	-1.2745
TCA cycle	PDHX	hsa-miR-135b	-1.2745
TCA cycle	PDHX	hsa-miR-181a	-0.1862
TCA cycle	PDHX	hsa-miR-181b	-0.1862
TCA cycle	PDHX	hsa-miR-181c	-0.1862
TCA cycle	PDHX	hsa-miR-181d	-0.1845
TCA cycle	PDHX	hsa-miR-182	-1.1407
TCA cycle	PDHX	hsa-miR-203	-1.086
TCA cycle	PDHX	hsa-miR-204	-1.2571
TCA cycle	PDHX	hsa-miR-211	-1.2571
TCA cycle	PDHX	hsa-miR-217	-0.3893
TCA cycle	PDHX	hsa-miR-23a	-1.1655
TCA cycle	PDHX	hsa-miR-23b	-1.1655
TCA cycle	PDHX	hsa-miR-26a	-1.2686
TCA cycle	PDHX	hsa-miR-26b	-1.2686
TCA cycle	PDHX	hsa-miR-27a	-1.1611
TCA cycle	PDHX	hsa-miR-27b	-1.1611
TCA cycle	PDHX	hsa-miR-29a	-0.7632
TCA cycle	PDHX	hsa-miR-29b	-0.7632
TCA cycle	PDHX	hsa-miR-29c	-0.7632
TCA cycle	PDHX	hsa-miR-300	-0.1545

TCA cycle	PDHX	hsa-miR-31	-1.2565
TCA cycle	PDHX	hsa-miR-335	-0.1533
TCA cycle	PDHX	hsa-miR-33a	-0.1791
TCA cycle	PDHX	hsa-miR-33b	-0.1791
TCA cycle	PDHX	hsa-miR-342-3p	-0.3945
TCA cycle	PDHX	hsa-miR-371-5p	-0.2574
TCA cycle	PDHX	hsa-miR-381	-0.1545
TCA cycle	PDHX	hsa-miR-382	-0.9638
TCA cycle	PDHX	hsa-miR-384	-0.1569
TCA cycle	PDHX	hsa-miR-410	-0.201
TCA cycle	PDHX	hsa-miR-494	-0.1856
TCA cycle	PDHX	hsa-miR-495	-0.2283
TCA cycle	PDHX	hsa-miR-543	-0.2187
TCA cycle	PDHX	hsa-miR-590-3p	-1.1881
TCA cycle	PDHX	hsa-miR-599	-0.3234
TCA cycle	PDHX	hsa-miR-96	-0.1663
TCA cycle	PDK1	hsa-miR-1271	-0.5616
TCA cycle	PDK1	hsa-miR-128	-0.5797
TCA cycle	PDK1	hsa-miR-130a	-0.4381
TCA cycle	PDK1	hsa-miR-130b	-0.4381
TCA cycle	PDK1	hsa-miR-136	-0.837
TCA cycle	PDK1	hsa-miR-138	-0.7625
TCA cycle	PDK1	hsa-miR-139-5p	-0.6253
TCA cycle	PDK1	hsa-miR-143	-0.6697
TCA cycle	PDK1	hsa-miR-148a	-0.499
TCA cycle	PDK1	hsa-miR-148b	-0.499
TCA cycle	PDK1	hsa-miR-152	-0.499
TCA cycle	PDK1	hsa-miR-155	-0.347
TCA cycle	PDK1	hsa-miR-181a	-0.351
TCA cycle	PDK1	hsa-miR-181b	-0.351
TCA cycle	PDK1	hsa-miR-181c	-0.3538
TCA cycle	PDK1	hsa-miR-181d	-0.351

TCA cycle	PDK1	hsa-miR-216a	-0.4515
TCA cycle	PDK1	hsa-miR-224	-0.1217
TCA cycle	PDK1	hsa-miR-25	-0.1206
TCA cycle	PDK1	hsa-miR-27a	-0.2225
TCA cycle	PDK1	hsa-miR-27b	-0.2225
TCA cycle	PDK1	hsa-miR-296-3p	-0.8276
TCA cycle	PDK1	hsa-miR-301a	-0.4457
TCA cycle	PDK1	hsa-miR-301b	-0.4457
TCA cycle	PDK1	hsa-miR-32	-0.1243
TCA cycle	PDK1	hsa-miR-320a	-0.6456
TCA cycle	PDK1	hsa-miR-320b	-0.6456
TCA cycle	PDK1	hsa-miR-320c	-0.9323
TCA cycle	PDK1	hsa-miR-320d	-0.6456
TCA cycle	PDK1	hsa-miR-339-5p	-0.2842
TCA cycle	PDK1	hsa-miR-340	-0.1052
TCA cycle	PDK1	hsa-miR-363	-0.1255
TCA cycle	PDK1	hsa-miR-367	-0.123
TCA cycle	PDK1	hsa-miR-374a	-0.9323
TCA cycle	PDK1	hsa-miR-374b	-0.9387
TCA cycle	PDK1	hsa-miR-379	-1.1644
TCA cycle	PDK1	hsa-miR-384	-0.2587
TCA cycle	PDK1	hsa-miR-454	-0.4392
TCA cycle	PDK1	hsa-miR-504	-0.1963
TCA cycle	PDK1	hsa-miR-9	-0.1495
TCA cycle	PDK1	hsa-miR-92a	-0.123
TCA cycle	PDK1	hsa-miR-92b	-0.123
TCA cycle	PDK1	hsa-miR-96	-0.5234
TCA cycle	SDHB	hsa-miR-411	-0.5718
TCA cycle	SDHB	hsa-miR-543	-0.7082
TCA cycle	SDHC	hsa-miR-129-5p	-0.3778
TCA cycle	SDHC	hsa-miR-140-5p	-0.398
TCA cycle	SDHC	hsa-miR-141	-0.6674

TCA cycle	SDHC	hsa-miR-146a	-0.7227
TCA cycle	SDHC	hsa-miR-146b-5p	-0.7227
TCA cycle	SDHC	hsa-miR-15a	-0.9175
TCA cycle	SDHC	hsa-miR-15b	-0.9207
TCA cycle	SDHC	hsa-miR-16	-0.9175
TCA cycle	SDHC	hsa-miR-185	-0.835
TCA cycle	SDHC	hsa-miR-195	-0.8916
TCA cycle	SDHC	hsa-miR-200a	-0.6674
TCA cycle	SDHC	hsa-miR-21	-0.2786
TCA cycle	SDHC	hsa-miR-216b	-0.3656
TCA cycle	SDHC	hsa-miR-223	-0.6625
TCA cycle	SDHC	hsa-miR-320a	-0.1634
TCA cycle	SDHC	hsa-miR-320b	-0.1634
TCA cycle	SDHC	hsa-miR-320c	-0.1634
TCA cycle	SDHC	hsa-miR-320d	-0.1634
TCA cycle	SDHC	hsa-miR-326	-0.1115
TCA cycle	SDHC	hsa-miR-330-5p	-0.1126
TCA cycle	SDHC	hsa-miR-365	-0.9017
TCA cycle	SDHC	hsa-miR-424	-0.9142
TCA cycle	SDHC	hsa-miR-491-5p	-0.5617
TCA cycle	SDHC	hsa-miR-495	-0.4413
TCA cycle	SDHC	hsa-miR-496	-1.2768
TCA cycle	SDHC	hsa-miR-497	-0.9142
TCA cycle	SDHC	hsa-miR-499-5p	-0.3573
TCA cycle	SDHC	hsa-miR-504	-0.4249
TCA cycle	SDHC	hsa-miR-542-3p	-0.4207
TCA cycle	SDHC	hsa-miR-551a	-0.2711
TCA cycle	SDHC	hsa-miR-551b	-0.2711
TCA cycle	SDHC	hsa-miR-590-5p	-0.2786
TCA cycle	SDHC	hsa-miR-7	-1.0115
TCA cycle	SDHD	hsa-miR-141	-1.1119
TCA cycle	SDHD	hsa-miR-200a	-1.1119

TCA cycle	SDHD	hsa-miR-204	-0.8734
TCA cycle	SDHD	hsa-miR-210	-0.2604
TCA cycle	SDHD	hsa-miR-211	-0.87
TCA cycle	SDHD	hsa-miR-216a	-1.1763
TCA cycle	SDHD	hsa-miR-216b	-0.1367
TCA cycle	SDHD	hsa-miR-23a	-1.152
TCA cycle	SDHD	hsa-miR-23b	-1.152
TCA cycle	SDHD	hsa-miR-320a	-0.9476
TCA cycle	SDHD	hsa-miR-320b	-0.9476
TCA cycle	SDHD	hsa-miR-320c	-0.9476
TCA cycle	SDHD	hsa-miR-320d	-0.9476
TCA cycle	SDHD	hsa-miR-361-5p	-0.1119
TCA cycle	SDHD	hsa-miR-371-5p	-1.0399
TCA cycle	SDHD	hsa-miR-374a	-1.1421
TCA cycle	SDHD	hsa-miR-374b	-1.1421
TCA cycle	SDHD	hsa-miR-376a	-1.3304
TCA cycle	SDHD	hsa-miR-376b	-1.3304
TCA cycle	SDHD	hsa-miR-382	-0.1346
TCA cycle	SDHD	hsa-miR-384	-0.6362
TCA cycle	SDHD	hsa-miR-410	-1.334
TCA cycle	SDHD	hsa-miR-433	-0.2804
TCA cycle	SDHD	hsa-miR-488	-1.0747
TCA cycle	SDHD	hsa-miR-496	-1.051
TCA cycle	SDHD	hsa-miR-543	-0.5852
TCA cycle	SDHD	hsa-miR-544	-1.2973
TCA cycle	SDHD	hsa-miR-590-3p	-1.2304
TCA cycle	SDHD	hsa-miR-758	-0.1017
TCA cycle	SDHD	hsa-miR-874	-0.1134
