# Supplementary material for: MicroRNAs and oncogenic transcriptional regulatory networks controlling metabolic reprogramming in cancers
Source: Comput Struct Biotechnol J. 2016 Jun 4;14:223–33. doi: 10.1016/j.csbj.2016.05.005 (PMC4915959; doi:10.1016/j.csbj.2016.05.005)
Supplement: Table S3 — miRNA-target gene interactions used to generate Fig. 4. [file mmc3.pdf]

Table S3

target genes	miRNAs	experimentally validation	The regulation of miRNA mediated by TFs
ACLY	hsa-miR-22	No	No
ACLY	hsa-miR-27a	No	No
ACLY	hsa-miR-27b	No	No
ALDOA	hsa-miR-329	No	No
ALDOA	hsa-miR-34a	No	No
ALDOA	hsa-miR-34c	No	No
ALDOA	hsa-miR-362	No	No
ALDOA	hsa-miR-449a	No	No
ALDOA	hsa-miR-449b	No	No
CIC	hsa-miR-1271	No	No
CIC	hsa-miR-96	No	No
FASN	hsa-miR-15a	No	No
FASN	hsa-miR-15b	No	No
FASN	hsa-miR-16	No	No
FASN	hsa-miR-195	No	No
FASN	hsa-miR-424	No	No
FASN	hsa-miR-497	No	No
G6PD	hsa-miR-613	No	No
GLS2	hsa-miR-15a	No	No
GLS2	hsa-miR-15b	No	No
GLS2	hsa-miR-16	No	No
GLS2	hsa-miR-195	No	No
GLS2	hsa-miR-424	No	No
GLS2	hsa-miR-497	No	No
GLUT1	hsa-miR-130a	No	No
GLUT1	hsa-miR-130b	No	No
GLUT1	hsa-miR-132	No	No
GLUT1	hsa-miR-140	No	No
GLUT1	hsa-miR-148a	No	No

GLUT1	hsa-miR-148b	No	No
GLUT1	hsa-miR-152	No	No
GLUT1	hsa-miR-19a	No	No
GLUT1	hsa-miR-19b	No	No
GLUT1	hsa-miR-212	No	No
GLUT1	hsa-miR-22	No	No
GLUT1	hsa-miR-301a	No	No
GLUT1	hsa-miR-301b	No	No
GLUT1	hsa-miR-328	No	No
GLUT1	hsa-miR-410	No	No
GLUT1	hsa-miR-454	No	No
GLUT2	hsa-miR-374a	No	No
GLUT2	hsa-miR-374b	No	No
GLUT3	hsa-miR-103	No	No
GLUT3	hsa-miR-107	No	No
GLUT3	hsa-miR-146a	No	No
GLUT3	hsa-miR-146b	No	No
GLUT3	hsa-miR-148a	No	No
GLUT3	hsa-miR-148b	No	No
GLUT3	hsa-miR-152	No	No
GLUT3	hsa-miR-15a	No	No
GLUT3	hsa-miR-15b	No	No
GLUT3	hsa-miR-16	No	No
GLUT3	hsa-miR-181a	No	No
GLUT3	hsa-miR-181b	No	No
GLUT3	hsa-miR-181c	No	No
GLUT3	hsa-miR-181d	No	No
GLUT3	hsa-miR-200b	No	No
GLUT3	hsa-miR-200c	No	No
GLUT3	hsa-miR-25	No	No
GLUT3	hsa-miR-29a	No	No
GLUT3	hsa-miR-29b	No	No

GLUT3	hsa-miR-29c	No	No
GLUT3	hsa-miR-32	No	No
GLUT3	hsa-miR-363	No	No
GLUT3	hsa-miR-367	No	No
GLUT3	hsa-miR-424	No	No
GLUT3	hsa-miR-429	No	No
GLUT3	hsa-miR-497	No	No
GLUT3	hsa-miR-542	No	No
GLUT3	hsa-miR-92a	No	No
GLUT3	hsa-miR-92b	No	No
GLUT4	hsa-miR-106a	No	No
GLUT4	hsa-miR-106b	No	No
GLUT4	hsa-miR-17	No	No
GLUT4	hsa-miR-20a	No	No
GLUT4	hsa-miR-20b	No	No
GLUT4	hsa-miR-302a	No	No
GLUT4	hsa-miR-302b	No	No
GLUT4	hsa-miR-302c	No	No
GLUT4	hsa-miR-302d	No	No
GLUT4	hsa-miR-302e	No	No
GLUT4	hsa-miR-31	No	No
GLUT4	hsa-miR-372	No	No
GLUT4	hsa-miR-373	No	No
GLUT4	hsa-miR-519d	No	No
GLUT4	hsa-miR-520a	No	No
GLUT4	hsa-miR-520b	No	No
GLUT4	hsa-miR-520c	No	No
GLUT4	hsa-miR-520d	No	No
GLUT4	hsa-miR-520e	No	No
GLUT4	hsa-miR-93	No	No
HK1	hsa-miR-138	No	No
HK1	hsa-miR-302a	No	No

HK1	hsa-miR-302b	No	No
HK1	hsa-miR-302c	No	No
HK1	hsa-miR-302d	No	No
HK1	hsa-miR-302e	No	No
HK1	hsa-miR-372	No	No
HK1	hsa-miR-373	No	No
HK1	hsa-miR-520a	No	No
HK1	hsa-miR-520b	No	No
HK1	hsa-miR-520c	No	No
HK1	hsa-miR-520d	No	No
HK1	hsa-miR-520e	No	No
HK2	hsa-miR-125a	No	No
HK2	hsa-miR-125b	No	No
HK2	hsa-miR-9	No	No
IDH1	hsa-miR-133a	No	No
IDH1	hsa-miR-133b	No	No
IDH1	hsa-miR-137	No	No
IDH1	hsa-miR-23a	No	No
IDH1	hsa-miR-23b	No	No
IDH1	hsa-miR-25	No	No
IDH1	hsa-miR-30a	No	No
IDH1	hsa-miR-30b	No	No
IDH1	hsa-miR-30c	No	No
IDH1	hsa-miR-30d	No	No
IDH1	hsa-miR-30e	No	No
IDH1	hsa-miR-32	No	No
IDH1	hsa-miR-363	No	No
IDH1	hsa-miR-367	No	No
IDH1	hsa-miR-92a	No	No
IDH1	hsa-miR-92b	No	No
IDH2	hsa-miR-144	No	No
LDHA	hsa-miR-33a	No	No

LDHA	hsa-miR-33b	No	No
LDHA	hsa-miR-34a	No	No
LDHA	hsa-miR-34c	No	No
LDHA	hsa-miR-383	No	No
LDHA	hsa-miR-449a	No	No
LDHA	hsa-miR-449b	No	No
MCT1	hsa-miR-154	No	No
MCT1	hsa-miR-216a	No	No
MCT1	hsa-miR-29a	No	No
MCT1	hsa-miR-29b	No	No
MCT1	hsa-miR-29c	No	No
MCT1	hsa-miR-342	No	No
MCT1	hsa-miR-374a	No	No
MCT1	hsa-miR-374b	No	No
MCT1	hsa-miR-384	No	No
MCT1	hsa-miR-506	No	No
MCT1	hsa-miR-539	No	No
ME1	hsa-miR-153	No	No
ME1	hsa-miR-30a	No	No
ME1	hsa-miR-30b	No	No
ME1	hsa-miR-30c	No	No
ME1	hsa-miR-30d	No	No
ME1	hsa-miR-30e	No	No
ME1	hsa-miR-448	No	No
MTHFD1L	hsa-miR-338	No	No
MTHFD1L	hsa-miR-377	No	No
MTHFD1L	hsa-miR-543	No	No
MTHFD2	hsa-miR-22	No	No
MTHFD2	hsa-miR-25	No	No
MTHFD2	hsa-miR-32	No	No
MTHFD2	hsa-miR-33a	No	No
MTHFD2	hsa-miR-33b	No	No

MTHFD2	hsa-miR-363	No	No
MTHFD2	hsa-miR-367	No	No
MTHFD2	hsa-miR-92a	No	No
MTHFD2	hsa-miR-92b	No	No
PDHX	hsa-miR-128	No	No
PDHX	hsa-miR-135a	No	No
PDHX	hsa-miR-135b	No	No
PDHX	hsa-miR-181a	No	No
PDHX	hsa-miR-181b	No	No
PDHX	hsa-miR-181c	No	No
PDHX	hsa-miR-181d	No	No
PDHX	hsa-miR-27a	No	No
PDHX	hsa-miR-27b	No	No
PDHX	hsa-miR-29a	No	No
PDHX	hsa-miR-29b	No	No
PDHX	hsa-miR-29c	No	No
PDK1	hsa-miR-1271	No	No
PDK1	hsa-miR-128	No	No
PDK1	hsa-miR-138	No	No
PDK1	hsa-miR-216a	No	No
PDK1	hsa-miR-27a	No	No
PDK1	hsa-miR-27b	No	No
PDK1	hsa-miR-96	No	No
PGAM1	hsa-miR-135a	No	No
PGAM1	hsa-miR-135b	No	No
PGAM1	hsa-miR-25	No	No
PGAM1	hsa-miR-32	No	No
PGAM1	hsa-miR-363	No	No
PGAM1	hsa-miR-367	No	No
PGAM1	hsa-miR-92a	No	No
PGAM1	hsa-miR-92b	No	No
PHGDH	hsa-miR-128	No	No

PSAT1	hsa-miR-145	No	No
PSAT1	hsa-miR-15a	No	No
PSAT1	hsa-miR-15b	No	No
PSAT1	hsa-miR-16	No	No
PSAT1	hsa-miR-195	No	No
PSAT1	hsa-miR-200b	No	No
PSAT1	hsa-miR-200c	No	No
PSAT1	hsa-miR-424	No	No
PSAT1	hsa-miR-429	No	No
PSAT1	hsa-miR-497	No	No
PSPH	hsa-miR-139	No	No
PSPH	hsa-miR-186	No	No
PSPH	hsa-miR-200b	No	No
PSPH	hsa-miR-200c	No	No
PSPH	hsa-miR-382	No	No
PSPH	hsa-miR-429	No	No
SCD	hsa-let-7a	No	No
SCD	hsa-let-7b	No	No
SCD	hsa-let-7c	No	No
SCD	hsa-let-7d	No	No
SCD	hsa-let-7e	No	No
SCD	hsa-let-7f	No	No
SCD	hsa-let-7g	No	No
SCD	hsa-let-7i	No	No
SCD	hsa-miR-124	No	No
SCD	hsa-miR-181a	No	No
SCD	hsa-miR-181b	No	No
SCD	hsa-miR-181c	No	No
SCD	hsa-miR-181d	No	No
SCD	hsa-miR-186	No	No
SCD	hsa-miR-200b	No	No
SCD	hsa-miR-200c	No	No

SCD	hsa-miR-216a	No	No
SCD	hsa-miR-382	No	No
SCD	hsa-miR-429	No	No
SCD	hsa-miR-495	No	No
SCD	hsa-miR-506	No	No
SCD	hsa-miR-98	No	No
SDHC	hsa-miR-140	No	No
SDHC	hsa-miR-185	No	No
SDHC	hsa-miR-216b	No	No
SDHC	hsa-miR-320a	No	No
SDHC	hsa-miR-320b	No	No
SDHC	hsa-miR-320c	No	No
SDHC	hsa-miR-320d	No	No
SDHC	hsa-miR-365	No	No
SDHC	hsa-miR-496	No	No
SDHD	hsa-miR-204	No	No
SDHD	hsa-miR-211	No	No
SDHD	hsa-miR-23a	No	No
SDHD	hsa-miR-23b	No	No
SDHD	hsa-miR-320a	No	No
SDHD	hsa-miR-320b	No	No
SDHD	hsa-miR-320c	No	No
SDHD	hsa-miR-320d	No	No
SHMT1	hsa-miR-218	No	No
SHMT1	hsa-miR-320a	No	No
SHMT1	hsa-miR-320b	No	No
SHMT1	hsa-miR-320c	No	No
SHMT1	hsa-miR-320d	No	No
SHMT1	hsa-miR-9	No	No
SHMT2	hsa-miR-149	No	No
SHMT2	hsa-miR-383	No	No
SHMT2	hsa-miR-485	No	No

SHMT2	hsa-miR-495	No	No
TKTL1	hsa-miR-203	No	No
G6PD	hsa-miR-1	Yes	No
G6PD	hsa-miR-206	Yes	No
GLUT3	hsa-miR-195	Yes	No
HK2	hsa-miR-143	Yes	No
IDH2	hsa-miR-183	Yes	No
MCT1	hsa-miR-124	Yes	No
MTHFD1L	hsa-miR-9	Yes	No
MTHFD2	hsa-miR-9	Yes	No
PKM2	hsa-miR-122	Yes	No
HK1	hsa-miR-22	Yes	HIF1α
HK1	hsa-miR-18a	Yes	HIF1α
HK1	hsa-miR-519c	Yes	HIF1α
HK1	hsa-miR-429	Yes	HIF1α
HK1	hsa-miR-17-92	Yes	HIF1α
HK1	hsa-miR-199a	Yes	HIF1α
HK2	hsa-miR-22	Yes	HIF1α
HK2	hsa-miR-18a	Yes	HIF1α
HK2	hsa-miR-519c	Yes	HIF1α
HK2	hsa-miR-429	Yes	HIF1α
HK2	hsa-miR-17-92	Yes	HIF1α
HK2	hsa-miR-199a	Yes	HIF1α
GLUT3	hsa-miR-22	Yes	HIF1α
GLUT3	hsa-miR-18a	Yes	HIF1α
GLUT3	hsa-miR-519c	Yes	HIF1α
GLUT3	hsa-miR-429	Yes	HIF1α
GLUT3	hsa-miR-17-92	Yes	HIF1α
GLUT3	hsa-miR-199a	Yes	HIF1α
ALDOA	hsa-miR-22	Yes	HIF1α
ALDOA	hsa-miR-18a	Yes	HIF1α
ALDOA	hsa-miR-519c	Yes	HIF1α

ALDOA	hsa-miR-429	Yes	HIF1α
ALDOA	hsa-miR-17-92	Yes	HIF1α
ALDOA	hsa-miR-199a	Yes	HIF1α
GLUT1	hsa-miR-22	Yes	HIF1α
GLUT1	hsa-miR-18a	Yes	HIF1α
GLUT1	hsa-miR-519c	Yes	HIF1α
GLUT1	hsa-miR-429	Yes	HIF1α
GLUT1	hsa-miR-17-92	Yes	HIF1α
GLUT1	hsa-miR-199a	Yes	HIF1α
PGK	hsa-miR-22	Yes	HIF1α
PGK	hsa-miR-18a	Yes	HIF1α
PGK	hsa-miR-519c	Yes	HIF1α
PGK	hsa-miR-429	Yes	HIF1α
PGK	hsa-miR-17-92	Yes	HIF1α
PGK	hsa-miR-199a	Yes	HIF1α
LDHA	hsa-miR-22	Yes	HIF1α
LDHA	hsa-miR-18a	Yes	HIF1α
LDHA	hsa-miR-519c	Yes	HIF1α
LDHA	hsa-miR-429	Yes	HIF1α
LDHA	hsa-miR-17-92	Yes	HIF1α
LDHA	hsa-miR-199a	Yes	HIF1α
PKM2	hsa-miR-22	Yes	HIF1α
PKM2	hsa-miR-18a	Yes	HIF1α
PKM2	hsa-miR-519c	Yes	HIF1α
PKM2	hsa-miR-429	Yes	HIF1α
PKM2	hsa-miR-17-92	Yes	HIF1α
PKM2	hsa-miR-199a	Yes	HIF1α
PDK1	hsa-miR-22	Yes	HIF1α
PDK1	hsa-miR-18a	Yes	HIF1α
PDK1	hsa-miR-519c	Yes	HIF1α
PDK1	hsa-miR-429	Yes	HIF1α
PDK1	hsa-miR-17-92	Yes	HIF1α

PDK1	hsa-miR-199a	Yes	HIF1α
MCT4	hsa-miR-22	Yes	HIF1α
MCT4	hsa-miR-18a	Yes	HIF1α
MCT4	hsa-miR-519c	Yes	HIF1α
MCT4	hsa-miR-429	Yes	HIF1α
MCT4	hsa-miR-17-92	Yes	HIF1α
MCT4	hsa-miR-199a	Yes	HIF1α
FASN	hsa-miR-451	Yes	c-MYC
FASN	hsa-miR-145	Yes	c-MYC
FASN	hsa-miR-135b	Yes	c-MYC
FASN	hsa-miR-34a	Yes	c-MYC
FASN	hsa-miR-34c	Yes	c-MYC
FASN	hsa-let-7a	Yes	c-MYC
FASN	hsa-let-7g	Yes	c-MYC
FASN	hsa-miR-320b	Yes	c-MYC
FASN	hsa-miR-155	Yes	c-MYC
FASN	hsa-miR-744	Yes	c-MYC
GLS1	hsa-miR-451	Yes	c-MYC
GLS1	hsa-miR-145	Yes	c-MYC
GLS1	hsa-miR-135b	Yes	c-MYC
GLS1	hsa-miR-34a	Yes	c-MYC
GLS1	hsa-miR-34c	Yes	c-MYC
GLS1	hsa-let-7a	Yes	c-MYC
GLS1	hsa-let-7g	Yes	c-MYC
GLS1	hsa-miR-320b	Yes	c-MYC
GLS1	hsa-miR-155	Yes	c-MYC
GLS1	hsa-miR-744	Yes	c-MYC
PDK1	hsa-miR-451	Yes	c-MYC
PDK1	hsa-miR-145	Yes	c-MYC
PDK1	hsa-miR-135b	Yes	c-MYC
PDK1	hsa-miR-34a	Yes	c-MYC
PDK1	hsa-miR-34c	Yes	c-MYC

PDK1	hsa-let-7a	Yes	c-MYC
PDK1	hsa-let-7g	Yes	c-MYC
PDK1	hsa-miR-320b	Yes	c-MYC
PDK1	hsa-miR-155	Yes	c-MYC
PDK1	hsa-miR-744	Yes	c-MYC
SHMT2	hsa-miR-451	Yes	c-MYC
SHMT2	hsa-miR-145	Yes	c-MYC
SHMT2	hsa-miR-135b	Yes	c-MYC
SHMT2	hsa-miR-34a	Yes	c-MYC
SHMT2	hsa-miR-34c	Yes	c-MYC
SHMT2	hsa-let-7a	Yes	c-MYC
SHMT2	hsa-let-7g	Yes	c-MYC
SHMT2	hsa-miR-320b	Yes	c-MYC
SHMT2	hsa-miR-155	Yes	c-MYC
SHMT2	hsa-miR-744	Yes	c-MYC
GLUT1	hsa-miR-451	Yes	c-MYC
GLUT1	hsa-miR-145	Yes	c-MYC
GLUT1	hsa-miR-135b	Yes	c-MYC
GLUT1	hsa-miR-34a	Yes	c-MYC
GLUT1	hsa-miR-34c	Yes	c-MYC
GLUT1	hsa-let-7a	Yes	c-MYC
GLUT1	hsa-let-7g	Yes	c-MYC
GLUT1	hsa-miR-320b	Yes	c-MYC
GLUT1	hsa-miR-155	Yes	c-MYC
GLUT1	hsa-miR-744	Yes	c-MYC
ALDOA	hsa-miR-451	Yes	c-MYC
ALDOA	hsa-miR-145	Yes	c-MYC
ALDOA	hsa-miR-135b	Yes	c-MYC
ALDOA	hsa-miR-34a	Yes	c-MYC
ALDOA	hsa-miR-34c	Yes	c-MYC
ALDOA	hsa-let-7a	Yes	c-MYC
ALDOA	hsa-let-7g	Yes	c-MYC

ALDOA	hsa-miR-320b	Yes	c-MYC
ALDOA	hsa-miR-155	Yes	c-MYC
ALDOA	hsa-miR-744	Yes	c-MYC
HK2	hsa-miR-451	Yes	c-MYC
HK2	hsa-miR-145	Yes	c-MYC
HK2	hsa-miR-135b	Yes	c-MYC
HK2	hsa-miR-34a	Yes	c-MYC
HK2	hsa-miR-34c	Yes	c-MYC
HK2	hsa-let-7a	Yes	c-MYC
HK2	hsa-let-7g	Yes	c-MYC
HK2	hsa-miR-320b	Yes	c-MYC
HK2	hsa-miR-155	Yes	c-MYC
HK2	hsa-miR-744	Yes	c-MYC
LDHA	hsa-miR-451	Yes	c-MYC
LDHA	hsa-miR-145	Yes	c-MYC
LDHA	hsa-miR-135b	Yes	c-MYC
LDHA	hsa-miR-34a	Yes	c-MYC
LDHA	hsa-miR-34c	Yes	c-MYC
LDHA	hsa-let-7a	Yes	c-MYC
LDHA	hsa-let-7g	Yes	c-MYC
LDHA	hsa-miR-320b	Yes	c-MYC
LDHA	hsa-miR-155	Yes	c-MYC
LDHA	hsa-miR-744	Yes	c-MYC
PKM2	hsa-miR-451	Yes	c-MYC
PKM2	hsa-miR-145	Yes	c-MYC
PKM2	hsa-miR-135b	Yes	c-MYC
PKM2	hsa-miR-34a	Yes	c-MYC
PKM2	hsa-miR-34c	Yes	c-MYC
PKM2	hsa-let-7a	Yes	c-MYC
PKM2	hsa-let-7g	Yes	c-MYC
PKM2	hsa-miR-320b	Yes	c-MYC
PKM2	hsa-miR-155	Yes	c-MYC

PKM2	hsa-miR-744	Yes	c-MYC
MTHFD2	hsa-miR-451	Yes	c-MYC
MTHFD2	hsa-miR-145	Yes	c-MYC
MTHFD2	hsa-miR-135b	Yes	c-MYC
MTHFD2	hsa-miR-34a	Yes	c-MYC
MTHFD2	hsa-miR-34c	Yes	c-MYC
MTHFD2	hsa-let-7a	Yes	c-MYC
MTHFD2	hsa-let-7g	Yes	c-MYC
MTHFD2	hsa-miR-320b	Yes	c-MYC
MTHFD2	hsa-miR-155	Yes	c-MYC
MTHFD2	hsa-miR-744	Yes	c-MYC
PHGDH	hsa-miR-451	Yes	c-MYC
PHGDH	hsa-miR-145	Yes	c-MYC
PHGDH	hsa-miR-135b	Yes	c-MYC
PHGDH	hsa-miR-34a	Yes	c-MYC
PHGDH	hsa-miR-34c	Yes	c-MYC
PHGDH	hsa-let-7a	Yes	c-MYC
PHGDH	hsa-let-7g	Yes	c-MYC
PHGDH	hsa-miR-320b	Yes	c-MYC
PHGDH	hsa-miR-155	Yes	c-MYC
PHGDH	hsa-miR-744	Yes	c-MYC
PSPH	hsa-miR-451	Yes	c-MYC
PSPH	hsa-miR-145	Yes	c-MYC
PSPH	hsa-miR-135b	Yes	c-MYC
PSPH	hsa-miR-34a	Yes	c-MYC
PSPH	hsa-miR-34c	Yes	c-MYC
PSPH	hsa-let-7a	Yes	c-MYC
PSPH	hsa-let-7g	Yes	c-MYC
PSPH	hsa-miR-320b	Yes	c-MYC
PSPH	hsa-miR-155	Yes	c-MYC
PSPH	hsa-miR-744	Yes	c-MYC
PSAT1	hsa-miR-451	Yes	c-MYC

PSAT1	hsa-miR-145	Yes	c-MYC
PSAT1	hsa-miR-135b	Yes	c-MYC
PSAT1	hsa-miR-34a	Yes	c-MYC
PSAT1	hsa-miR-34c	Yes	c-MYC
PSAT1	hsa-let-7a	Yes	c-MYC
PSAT1	hsa-let-7g	Yes	c-MYC
PSAT1	hsa-miR-320b	Yes	c-MYC
PSAT1	hsa-miR-155	Yes	c-MYC
PSAT1	hsa-miR-744	Yes	c-MYC
SHMT1	hsa-miR-451	Yes	c-MYC
SHMT1	hsa-miR-145	Yes	c-MYC
SHMT1	hsa-miR-135b	Yes	c-MYC
SHMT1	hsa-miR-34a	Yes	c-MYC
SHMT1	hsa-miR-34c	Yes	c-MYC
SHMT1	hsa-let-7a	Yes	c-MYC
SHMT1	hsa-let-7g	Yes	c-MYC
SHMT1	hsa-miR-320b	Yes	c-MYC
SHMT1	hsa-miR-155	Yes	c-MYC
SHMT1	hsa-miR-744	Yes	c-MYC
ACC1	hsa-miR-185	Yes	SREBP1
ACC1	hsa-miR-342	Yes	SREBP1
ACLY	hsa-miR-185	Yes	SREBP1
ACLY	hsa-miR-342	Yes	SREBP1
FASN	hsa-miR-185	Yes	SREBP1
FASN	hsa-miR-342	Yes	SREBP1
G6PD	hsa-miR-33	Yes	p53
G6PD	hsa-miR-504	Yes	p53
G6PD	hsa-miR-25	Yes	p53
G6PD	hsa-miR-125b	Yes	p53
G6PD	hsa-miR-125a	Yes	p53
G6PD	hsa-miR-1285	Yes	p53
G6PD	hsa-miR-30d	Yes	p53

G6PD	hsa-miR-380	Yes	p53
PCK1	hsa-miR-33	Yes	p53
PCK1	hsa-miR-504	Yes	p53
PCK1	hsa-miR-25	Yes	p53
PCK1	hsa-miR-125b	Yes	p53
PCK1	hsa-miR-125a	Yes	p53
PCK1	hsa-miR-1285	Yes	p53
PCK1	hsa-miR-30d	Yes	p53
PCK1	hsa-miR-380	Yes	p53
ME1	hsa-miR-33	Yes	p53
ME1	hsa-miR-504	Yes	p53
ME1	hsa-miR-25	Yes	p53
ME1	hsa-miR-125b	Yes	p53
ME1	hsa-miR-125a	Yes	p53
ME1	hsa-miR-1285	Yes	p53
ME1	hsa-miR-30d	Yes	p53
ME1	hsa-miR-380	Yes	p53
MCT1	hsa-miR-33	Yes	p53
MCT1	hsa-miR-504	Yes	p53
MCT1	hsa-miR-25	Yes	p53
MCT1	hsa-miR-125b	Yes	p53
MCT1	hsa-miR-125a	Yes	p53
MCT1	hsa-miR-1285	Yes	p53
MCT1	hsa-miR-30d	Yes	p53
MCT1	hsa-miR-380	Yes	p53
ACC1	hsa-miR-33	Yes	p53
ACC1	hsa-miR-504	Yes	p53
ACC1	hsa-miR-25	Yes	p53
ACC1	hsa-miR-125b	Yes	p53
ACC1	hsa-miR-125a	Yes	p53
ACC1	hsa-miR-1285	Yes	p53
ACC1	hsa-miR-30d	Yes	p53

GLUT3	hsa-miR-33	Yes	p53
GLUT3	hsa-miR-504	Yes	p53
GLUT3	hsa-miR-25	Yes	p53
GLUT3	hsa-miR-125b	Yes	p53
GLUT3	hsa-miR-125a	Yes	p53
GLUT3	hsa-miR-1285	Yes	p53
GLUT3	hsa-miR-30d	Yes	p53
GLUT3	hsa-miR-380	Yes	p53
ACLY	hsa-miR-33	Yes	p53
ACLY	hsa-miR-504	Yes	p53
ACLY	hsa-miR-25	Yes	p53
ACLY	hsa-miR-125b	Yes	p53
ACLY	hsa-miR-125a	Yes	p53
ACLY	hsa-miR-1285	Yes	p53
ACLY	hsa-miR-30d	Yes	p53
ACLY	hsa-miR-380	Yes	p53
PGAM1	hsa-miR-33	Yes	p53
PGAM1	hsa-miR-504	Yes	p53
PGAM1	hsa-miR-25	Yes	p53
PGAM1	hsa-miR-125b	Yes	p53
PGAM1	hsa-miR-125a	Yes	p53
PGAM1	hsa-miR-1285	Yes	p53
PGAM1	hsa-miR-30d	Yes	p53
PGAM1	hsa-miR-380	Yes	p53
FASN	hsa-miR-33	Yes	p53
FASN	hsa-miR-504	Yes	p53
FASN	hsa-miR-25	Yes	p53
FASN	hsa-miR-125b	Yes	p53
FASN	hsa-miR-125a	Yes	p53
FASN	hsa-miR-1285	Yes	p53
FASN	hsa-miR-30d	Yes	p53
FASN	hsa-miR-380	Yes	p53

GLUT4	hsa-miR-33	Yes	p53
GLUT4	hsa-miR-504	Yes	p53
GLUT4	hsa-miR-25	Yes	p53
GLUT4	hsa-miR-125b	Yes	p53
GLUT4	hsa-miR-125a	Yes	p53
GLUT4	hsa-miR-1285	Yes	p53
GLUT4	hsa-miR-30d	Yes	p53
GLUT4	hsa-miR-380	Yes	p53
GLUT1	hsa-miR-33	Yes	p53
GLUT1	hsa-miR-504	Yes	p53
GLUT1	hsa-miR-25	Yes	p53
GLUT1	hsa-miR-125b	Yes	p53
GLUT1	hsa-miR-125a	Yes	p53
GLUT1	hsa-miR-1285	Yes	p53
GLUT1	hsa-miR-30d	Yes	p53
GLUT1	hsa-miR-380	Yes	p53
ALDOA	hsa-miR-33	Yes	p53
ALDOA	hsa-miR-504	Yes	p53
ALDOA	hsa-miR-25	Yes	p53
ALDOA	hsa-miR-125b	Yes	p53
ALDOA	hsa-miR-125a	Yes	p53
ALDOA	hsa-miR-1285	Yes	p53
ALDOA	hsa-miR-30d	Yes	p53
ALDOA	hsa-miR-380	Yes	p53
HK1	hsa-miR-33	Yes	p53
HK1	hsa-miR-504	Yes	p53
HK1	hsa-miR-25	Yes	p53
HK1	hsa-miR-125b	Yes	p53
HK1	hsa-miR-125a	Yes	p53
HK1	hsa-miR-1285	Yes	p53
HK1	hsa-miR-30d	Yes	p53
HK1	hsa-miR-380	Yes	p53

PDK2	hsa-miR-33	Yes	p53
PDK2	hsa-miR-504	Yes	p53
PDK2	hsa-miR-25	Yes	p53
PDK2	hsa-miR-125b	Yes	p53
PDK2	hsa-miR-125a	Yes	p53
PDK2	hsa-miR-1285	Yes	p53
PDK2	hsa-miR-30d	Yes	p53
PDK2	hsa-miR-380	Yes	p53
ME2	hsa-miR-33	Yes	p53
ME2	hsa-miR-504	Yes	p53
ME2	hsa-miR-25	Yes	p53
ME2	hsa-miR-125b	Yes	p53
ME2	hsa-miR-125a	Yes	p53
ME2	hsa-miR-1285	Yes	p53
ME2	hsa-miR-30d	Yes	p53
ME2	hsa-miR-380	Yes	p53
HK2	hsa-miR-33	Yes	p53
HK2	hsa-miR-504	Yes	p53
HK2	hsa-miR-25	Yes	p53
HK2	hsa-miR-125b	Yes	p53
HK2	hsa-miR-125a	Yes	p53
HK2	hsa-miR-1285	Yes	p53
HK2	hsa-miR-30d	Yes	p53
HK2	hsa-miR-380	Yes	p53
GLS2	hsa-miR-33	Yes	p53
GLS2	hsa-miR-504	Yes	p53
GLS2	hsa-miR-25	Yes	p53
GLS2	hsa-miR-125b	Yes	p53
GLS2	hsa-miR-125a	Yes	p53
GLS2	hsa-miR-1285	Yes	p53
GLS2	hsa-miR-30d	Yes	p53
GLS2	hsa-miR-380	Yes	p53

PDHX	hsa-miR-33	Yes	p53
PDHX	hsa-miR-504	Yes	p53
PDHX	hsa-miR-25	Yes	p53
PDHX	hsa-miR-125b	Yes	p53
PDHX	hsa-miR-125a	Yes	p53
PDHX	hsa-miR-1285	Yes	p53
PDHX	hsa-miR-30d	Yes	p53
PDHX	hsa-miR-380	Yes	p53
GLUT1	hsa-miR-1291	Yes	No
GLUT3	hsa-miR-106	Yes	No
PKM2	hsa-miR-133a	Yes	No
PKM2	hsa-miR-133b	Yes	No
PKM2	hsa-miR-326	Yes	No
LDHA	hsa-miR-21	Yes	No
TKTL1	hsa-miR-206	Yes	No
TKTL1	hsa-miR-1	Yes	No
PDHX	hsa-miR-26a	Yes	No
SDHD	hsa-miR-210	Yes	No
GLS1	hsa-miR-23a	Yes	No
GLS1	hsa-miR-23b	Yes	No
GLS2	hsa-miR-23a	Yes	No
GLS2	hsa-miR-23b	Yes	No
SHMT1	hsa-miR-198	Yes	No
SHMT2	hsa-miR-193b	Yes	No
PSAT1	hsa-miR-340	Yes	No
FASN	hsa-miR-320	Yes	No
PLD1	hsa-miR-638	Yes	No
